# Synthesis of Alkyl α-Amino-benzylphosphinates by the Aza-Pudovik Reaction; The Preparation of the Butyl Phenyl-*H*-phosphinate Starting P-Reagent

**DOI:** 10.3390/molecules30020339

**Published:** 2025-01-16

**Authors:** Bence Bajusz, Dorka Nagy, Regina Tóth, Zsuzsanna Szalai, Ágnes Gömöry, Angéla Takács, László Kőhidai, György Keglevich

**Affiliations:** 1 Department of Organic Chemistry and Technology, Faculty of Chemical Technology and Biotechnology, Budapest University of Technology and Economics, Műegyetem rkp. 3, 1111 Budapest, Hungary; bajusz.bence@edu.bme.hu (B.B.); nagydorka0801@gmail.com (D.N.); regina@icont.hu (R.T.); sz.zsuzsi97@gmail.com (Z.S.); 2 MS Proteomics Research Group, Research Centre for Natural Sciences, 1117 Budapest, Hungary; gomory.agnes@ttk.hu; 3 Department of Genetics, Cell- and Immunobiology, Semmelweis University, Nagyvárad tér 4, 1089 Budapest, Hungary; takacs.angela@semmelweis.hu (A.T.); kohlasz2@gmail.com (L.K.)

**Keywords:** alkyl phenyl-*H*-phosphinates, aza-Pudovik reaction, Kabachnik–Fields reaction, α-aminophosphinates, microwave, cytotoxic activity

## Abstract

Butyl phenyl-*H*-phosphinate that is not available commercially was prepared from phenyl-*H*-phosphinic acid by three methods: by alkylating esterification (i), by microwave-assisted direct esterification (ii), and unexpectedly, by thermal esterification (iii). Considering the green aspects, selectivity and scalability, the thermal variation seemed to be optimal. However, there was need for prolonged heating. The butyl phenyl-*H*-phosphinate, along with the ethyl analogue, was utilized in the synthesis of alkyl (α-alkylamino-arylmethyl-)phenyl phosphinates in the aza-Pudovik reaction with imines obtained from primary amines and substituted benzaldehydes. The aminophosphinates were obtained as diastereomeric mixtures in 65–92% yields. The aza-Pudovik approach was more efficient than the Kabachnik–Fields condensation. Interestingly, one aminophosphinate, the butyl (α-butylamino-benzyl-)phenylphosphinate, was of significant cytotoxic activity on the PANC-1 pancreas cell line. Another derivative, ethyl (α-benzylamino-benzyl-)phenylphosphinate, revealed a selective toxic activity on U266 myeloma cells.

## 1. Introduction

α-Aminophosphinic derivatives represent a hot topic, on the one hand, because of their biological activity, which is connected with their enzyme inhibitory properties [1,2,3,4,5,6,7,8,9,10,11,12,13], and on the other hand, as their synthesis poses challenges [14,15,16]. The basic method for the preparation of α-aminophosphonates is the three-component reaction of an oxo compound, an amine and a dialkyl phosphite. The condensation serves as an excellent model for green chemical investigations. A number of catalysts were suggested to promote the phospha-Mannich condensation [17,18,19,20,21,22,23,24,25,26,27,28,29,30,31,32]; however, it was shown by Keglevich and his colleague that under microwave (MW) irradiation, in most cases, there is no need for any catalyst [33]. Hence, cost and environmental burdens may be saved. Moreover, very often, the MW-assisted variations may be performed in a solvent-free manner. α-Aminophosphonates may be modified further by acylation [34], or by another phospha-Mannich condensation. This latter one, which is a tandem Kabachnik–Fields reaction, depending on the reagents, may lead to symmetrical or unsymmetrical [34] bis(phosphonoalkyl)amines. It turned out that the modified α-aminophosphonates may be of considerable cytotoxic activity on human breast and prostate adenocarcinoma, as well as on lung and epidermoid carcinoma [35]. It was found that even small structural changes may have a significant effect on the biological activity. The data accumulated on the biological effects of different phosphonates in the literature have been surveyed. The aza-Pudovik addition is another attractive approach for the synthesis of α-aminophosphonates [14]. According to this, a dialkyl phosphite may be easily added on the C=N unit of imines. This is a clear-cut and fully atomic efficient approach towards α-aminophosphonates. As a matter of fact, the imines may be intermediates in the phospha-Mannich reaction. The third possibility for the preparation of α-aminophosphonates is the MW-assisted nucleophilic substitution on the α-carbon atom of α-hydroxyphosphonates by amines, which is possible due to the beneficial effect of the adjacent P=O group, as proved by quantum chemical calculations. In our earlier study, we investigated the Kabachnik–Fields condensation of a few alkyl phenyl-*H*-phosphinates, formaldehyde and a few amines [36].

In this study, we wish to extend this reaction using different oxo-compounds, such as benzaldehyde and its substituted derivatives. It was also a challenge to investigate the possible ways for the preparation of the starting P-reagent, butyl phenyl-*H*-phosphinate. Pre-experiments, in order to synthesize the butyl ester by alkylating of the phenyl-*H*-phosphinic acid, were available [36]. Preparation of the target ester by the MW-assisted direct esterification of phenyl-*H*-phosphinic acid with butyl alcohol was also described by us; however, the size of these transformations was rather limited [37]. On the one hand, our purpose was to elaborate an optimized and scaled up synthesis of butyl phenyl-*H*-phosphinate; on the other hand, we wished to prepare new alkyl (α-alkylamino-arylmethyl-)phenylphosphinates either by the aza-Pudovik or the Kabachnik–Fields approach. Lastly, we planned to evaluate the cytotoxic effect of a few aminophosphinates made available on two cell lines.

## 2. Results and Discussion

### 2.1. A Study on the Preparation of Butyl Phenyl-H-phosphinate

**Scheme 1:**
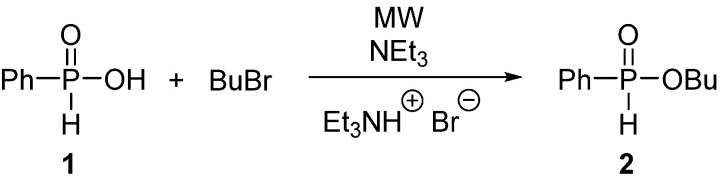
Alkylating esterification of phenyl-*H*-phosphinic acid with butyl bromide.

Phenyl-*H*-phosphinic acid (**1**) was reacted with 1.2 equiv. of butyl bromide in the presence of 1 equiv. of triethylamine under MW irradiation (Scheme 1). After an irradiation at 80 °C for 1.5 h, the conversion was only 46%. At 100 °C, a complete conversion was attained after 3.25 h. Butyl phenyl-*H*-phosphinate (**2**) was obtained in 76% yield after flash column chromatography.

The next option was the direct esterification of phenyl-*H*-phosphinic acid **1**, meaning its reaction with butyl alcohol. It is known that heteroatomic acids, such as phosphinic acids, resist undergoing esterification. However, it was found by Keglevich and Kiss that under MW irradiation, esterifications take place. It was a further development that suitable imidazolyum (e.g., bmim) salts applied in a quantity of 10% promoted the formation of phosphinates [38]. The ionic liquid had a dual role; it served as a MW absorber and as a catalyst [39]. So, the reaction of phosphinic acid with butyl alcohol applied in a 15-fold quantity took place at 140 °C under MW conditions. The butyl ester (**2**) was obtained in a yield of 94% [37]. The low capacity (0.10 g phosphinate **2** after a 30 min run) of the MW batch esterification approach made for an obstacle to further utilizations. The flow accomplishment provided somewhat better productivity (ca. 0.75 g ester **2** after 30 min) [37].

**Scheme 2:**
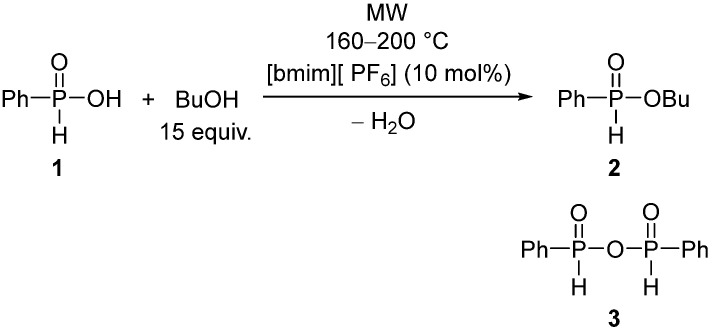
MW-assisted direct esterification of phenyl-*H*-phosphinic acid with butyl alcohol.

In the hope of better results, we decided to study further the MW-assisted [bmim][PF_6_]-promoted esterification of phosphinic acid (**1**) with butyl alcohol (Scheme 2) in order to try to perform it on a larger scale. The MW-assisted esterifications, starting from 0.70 mmol of phosphinic acid **1** and using 15-fold butyl alcohol along with 10% of [bmim][PF_6_], were performed at 160 °C for 1.5 h. The yield of butyl ester **2** was 80% after flash column chromatography (Table 1/Entry 1). Then, the reaction was set on a 2-fold scale. After an irradiation of 2.5 h, the conversion was 86%. There was 77% of ester **2**, and surprisingly, 9% of diphenyl pyrophosphinate (**3**) in the crude mixture (Table 1/Entry 2). Repeating the run at 180 °C for 1.5 h, the conversion was 78% with 69% of the ester (**2**) and 9% of by-product **3** (Table 1/Entry 3). Carrying out the esterification on a 4-fold scale at 185 °C/3.5 h and 200 °C/4 h, the conversions were even lower, 59% and 50%, respectively, exhibiting the ester (**2**) in 54% and 45%, respectively, with ca. 5/5% pyrophosphinate (**3**) present (Table 1/entries 4 and 5).

One can see that the MW accomplishment of the esterification under discussion is not suitable for scaling up. On the one hand, prolonged irradiation times may favor the decomposition of the butyl phosphinate (**2**) by hydrolysis. On the other hand, higher temperatures do not allow a complete shift of the equilibrium toward the phosphinate (**2**). On top of that, in the larger batches, there was a greater inclination for the formation of pyrophosphinate (**3**). By-product **3** is formed by the nucleophilic attack of the OH group of unreacted phosphinic acid **1** on the P=O unit of ester **2**. One may see that the batch MW-assisted method is not suitable for the preparation of butyl phenyl-*H*-phosphinate (**2**) on a larger scale.

**Scheme 3:**
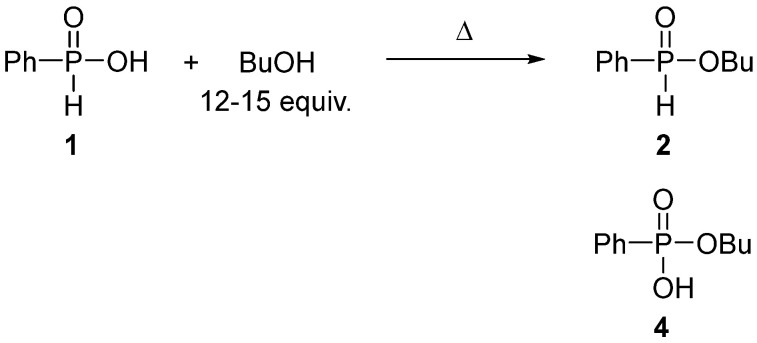
Thermal esterification of phenyl-*H*-phosphinic acid **1** with butyl alcohol.

For this, we risked trying the simple thermal esterification of 1.4 mmol phenylphosphinic acid **1** with 15-fold butyl alcohol under reflux conditions (Scheme 3). After 36 h, to our surprise, the reaction was practically complete (Table 2/Entry 1). Repeating the esterification on a 10-fold scale and applying butyl alcohol in a 12-fold quantity, we were again successful, as the major component (86%) was phosphinate **2**, and only 5% of starting material **1** was present. At the same time, the oxidized derivative (**4**) of the phosphinate (**2**) also appeared as a minor (9%) component (Table 2/Entry 2). Obviously, an O atom was inserted into the P–H bond under aerobic conditions to furnish phosphonic derivative PhP(O)(OBu)(OH) (**4**). To avoid the formation of this minor by-product, the experiment was repeated on a smaller scale in a glass “bomb”, but before closing the vessel, the system was inertized by bubbling N_2_ through the mixture. Sure enough, after a treatment at ~110 °C for 72 h, only the desired ester (**2**) was present in the mixture (Table 2/Entry 3). Repeating the large-scale esterification under N_2_ atmosphere, the conversion was 100%, and the yield of ester **2** was 95% after flash chromatography. However, there was need for a prolonged reflux of 60 h (Table 2/Entry 4). To decrease the reaction time, the thermal variation was also performed, applying 10% of [bmim][PF_6_] as the catalyst. Sure enough, the reaction time needed to be decreased to 30 h (Table 2/Entry 5).

It can be concluded that if butyl phenyl-*H*-phosphinate (**2**) has to be prepared on a g-scale, the best method is to reflux phosphinic acid **1** in 12-fold butyl alcohol under an inert atmosphere in the presence of 10% of [bmim][PF_6_]. Butyl phenyl-*H*-phosphinate **2** and by-products **3** and **4** were identified by ^31^P NMR and MS (Table 3).

### 2.2. Synthesis of Alkyl (α-Alkylamino-arylmethyl-)phenylphosphinates

**Scheme 4:**
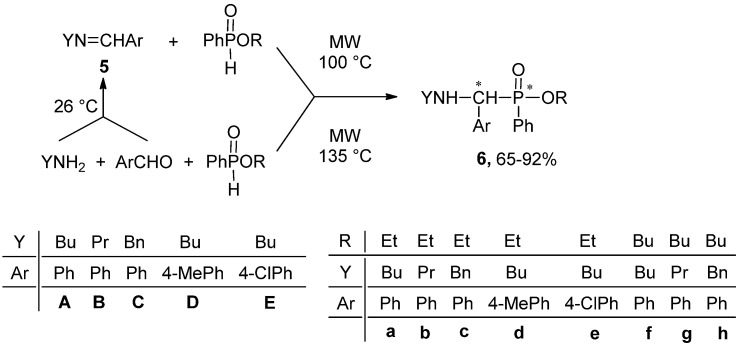
Synthesis of α-aminophosphinates by the aza-Pudovik reaction and by the Kabachnik–Fields condensation. *: Chirality centers.

The title compounds (**6**) may be prepared by the Kabachnik–Fields condensation of primary amines, benzaldehyde derivates and alkyl phenyl-*H*-phosphinates, or by the Pudovik reaction involving the addition of the >P(O)H reagent on the C=N unit of imines (**5**) (Scheme 4).

In the first approach, we applied the aza-Pudovik reaction. In the first step, butyl-, propyl- and benzylamine were reacted with benzaldehyde at 26 °C for 1 h to afford the corresponding imines (**5A**–**C**), whose interaction with ethyl phenyl-*H*-phosphinate at 100 °C for 1.5 h under MW irradiation afforded α-amino-benzylphosphinates **6a**–**c**. As an extension, the imines derived from 4-methyl-benzaldehyde and 4-chloro-benzaldehyde (**5D** and **5E**) were also involved in the aza-Pudovik reaction with the ethyl phosphinate, and imines **5A**–**C** were reacted with butyl phenyl-*H*-phosphinate as well. Addition of the racemic P-reagent on the prochiral C=N function of the imines **5A**–**C** afforded the aminophosphinic ester (**6a**–**h**) in all cases as a 1:1 mixture of diastereoisomers. On purification by column chromatography, in most cases, the main fraction was enriched in one of the isomers. The isomeric mixtures isolated in 65–92% yields were characterized by ^31^P, ^13^C and ^1^H NMR, as well as HRMS. Only aminohosphinates **6a** and **6f** were also prepared by the Kabachnik–Fields condensation. On the basis of the yields (70/78% vs. 54/75%), the preparation via the aza-Pudovik route seemed to be more efficient.

It should be noted that a series of new ethyl or butyl (α-alkylamino-arylmethyl-)phenylphosphinates, representing a brand new family of phosphinates, were synthesized, due to the two chiral centers, as a mixture of two diastereomers. As marked by the 65–92% yields, the aza-Pudovik route seemed to be somewhat more efficient than the three-component Kabachnik–Field condensation.

### 2.3. Cytotoxic Activity of a Few of Alkyl (α-Alkylamino-arylmethyl-)phenylphosphinates

The aminophosphinates prepared were tested on PANC-1 pancreas and U266 myeloma cancer cell lines in three different doses. In our research group, testing the new phosphonate and related derivative against bone diseases (e.g., on myeloma cancer cell lines) is an emphasized purpose. Butyl (α-butylamino-benzyl-)phenylphosphinate (**6f**) demonstrated the most potent antiproliferative activity on PANC-1 cells at 100 μM, reducing cell viability to 30% of control. This aminophosphinate (**6f**) also demonstrated a significant reduction in the viability of U266 cells at 10 μM, lowering viability to 81%. Interestingly, ethyl (α-benzylamino-benzyl-)phenylphosphinate **6c** exhibited a selective activity, significantly reducing U266 viability, but only at 1 μM. The other aminophosphinates (**6a**,**b**,**d**,**e**) had minimal or no significant effects at neither investigated concentrations on both cell lines (Table 4).

To the best of our knowledge, no cytotoxic data of α-aminophosphinates on PANC-1 and U266 cell lines have been previously published. At the same time, the cytotoxic effect of different α-aminophosphonic derivatives was evaluated by us. According to this, a part of the *N*-acyl- and *N*-phosphinoylmetyl- or *N*-phosphonoylmethyl derivatives revealed a significant effect on human breast adenocarcinoma, prostate adenocarcinoma, lung carcinoma (Ebc-1) and epidermoid carcinoma (A431) cells [35]. Cytostasis values >50% were measured on Ebc-1 and A431 cell cultures to the effect of an *N*-diarylphosphinoylmetyl-α-amino-benzylphosphonate applied in 50 μM doses.

## 3. Materials and Methods (Experiment)

### 3.1. General

The ^31^P, ^13^C, ^1^H-NMR spectra were taken on a Bruker DRX-500 spectrometer (Bruker, Billerica, MA, USA) operating at 202.4, 125.7, and 500 MHz. The couplings are given in Hz. HPLC-MS measurements were performed using a Shimadzu LCMS-2020 device (Shimadzu Corporation, Kioto, Japan) equipped with a Reprospher 100 C18 (Dr. Maisch, Ammerbuch-Entringen, Germany) (5 μm; 100 × 3 mm) column and positive–negative double ion source (DUIS±) with a quadrupole MS analysator in a range of 50–1000 *m*/*z*. The sample was eluted with gradient elution using eluent A (0.1% formic acid in water) and eluent B (0.1% formic acid in acetonitrile). The flow rate was set to 1.5 mL/min. The column temperature was kept at room temperature and the injection volume was 1–10 μL. The purity of compounds was assessed by HPLC with UV detection at 215 and 254 nm. High-resolution mass spectrometric measurements were performed using a Thermo Velos Pro Orbitrap Elite hybrid mass spectrometer (Thermo Fisher Scientific, Waltham, MA, USA) in positive electrospray mode.

### 3.2. The Preparation of Butyl-phenyl-H-phosphinates

#### 3.2.1. Alkylating Esterification

A mixture of 0.28 g (2.0 mmol) of phenyl-*H*-phosphinic acid (**1**), 0.26 mL (2.4 mmol) of butyl bromide and 0.28 mL (2.0 mmol) of triethylamine in a glass vessel was irradiated in a CEM Discover microwave reactor equipped with a pressure-controller at 100 °C for 3.25 h. The excess butyl bromide was removed under vacuum, and the crude product so obtained was purified by column chromatography using silica gel absorbent and ethyl acetate eluent to afford 0.21 g (76%) of ester **2** as a colorless oil.

#### 3.2.2. MW-Assisted Esterification

Quantities of 0.20 g (1.4 mmol) of phenyl-*H*-phosphinic acid (**1**), 2.0 mL (21.0 mmol) of butyl alcohol and 29 μL (0.14 mmol) of [bmim][PF_6_] were measured into the reaction vessel, then the resulting mixture was irradiated in a CEM microwave reactor equipped with a pressure-controller at 160 °C for 2.5 h. The excess butyl alcohol was removed under vacuum, and the resulting crude product was purified by column chromatography (as above) to give 0.14 g (71%) of ester **2** as a colorless oil.

#### 3.2.3. Thermal Esterification

A mixture of 0.50 g (3.5 mmol) of phenyl-*H*-phosphinic acid (**1**), 3.8 mL (42.0 mmol) of butyl alcohol and 72 μL (0.35 mmol) of [bmim][PF_6_] was inertized, and then stirred at the boiling point under nitrogen under conventional (oil bath) heating for 26 h. The excess butyl alcohol was removed under vacuum, and the crude product so obtained was purified by column chromatography (as above) to furnish 0.55 g (80%) of ester **2** as a colorless oil.

### 3.3. The Preparation of Alkoxy α-Alkylamino-benzyl-phenylphosphinates

#### 3.3.1. General Procedure for the Synthesis of Alkoxy α-Alkylamino-benzyl-phenylphosphinates (**6a**–**h**) by the aza-Pudovik Reaction

The mixture of 25 mmol of the aldehyde (benzaldehyde: 2.6 mL, 4-chlorobenzaldehyde: 3.5 g, 4-methyl-benzaldehyde: 2.94 mL), and 25 mmol of the amine (butylamine: 2.5 mL, propylamine: 2.1 mL, benzylamine: 2.7 mL) was stirred for 1 h at room temperature, then 10 mL of dichloromethane was added and the water formed was removed by adding 10 g of Na_2_SO_4_. After filtration, the solvent was removed, and the residue purified by flash chromatography (silica gel, dichloromethane:methanol (97:3) as the eluent).

In the second step, 1.0 mmol of the above-synthetized imine [*N*-benzylidenepropylimine: 0.15 g, *N*-benzylidenebutylimine: 0.16 g, *N*-benzylidenebenzylimine: 0.20 g, *N*-(4-chlorobenzylidene)-buthylimine: 0.20 g, *N*-(4-methylbenzylidene)-buthylimine: 0.18 g] and 1.5 mmol phosphinate [ethyl-phenyl-H-phosphinate: 0.25 mL, butyl-phenyl-*H*-phosphinate: 0.30 g] were added to the MW glass vessel. Then, the vial was closed, inserted in the MW reactor, and irradiated isothermally at 100 °C for 1.5 h. The reaction mixture was purified by column chromatography (silica gel, dichloromethane:methanol (97:3)/acetone:hexane (8:2) as the eluent) to afford products **6a**–**h**. Yields: **6a**: 70%, **6b**: 67%, **6c**: 65%, **6d**: 71%, **6e**: 69%, **6f**: 78%, **6g**: 88% and **6h**: 92%.

The following products were thus prepared:

**6a-1**: ^31^P NMR (CDCl_3_) δ 38.1 (74%); ^13^C NMR (CDCl_3_) 13.87 (s, CH_3_CH_2_CH_2_), 16.4 (d, *J* = 5.9 Hz, CH_3_CH_2_O), 20.2 (s, CH_3_CH_2_CH_2_), 31.75 (s, CH_2_CH_2_NH), 47.7 (d, *J* = 14.7 Hz, CH_2_N), 61.5 (d, *J* = 6.8 Hz, CH_2_O), 63.94 (d, *J* = 107.6 Hz, CHPh), 127.6 (d, *J* = 3.1 Hz, δ)^a^, 128.0 (d, *J* = 11.9 Hz, β′), 128.09 (s, γ′), 128.8 (d, *J* = 5.4 Hz, β)^b^, 129.4 (d, *J* = 126.5 Hz, α), 132.2 (d, *J* = 2.8 Hz, δ′)^a^, 132.4 (d, *J* = 9.3 Hz, γ)^b^, 135.6 (bs, α′), ^a,b^ may be reversed.

**6a-2**: ^31^P NMR (CDCl_3_) δ 39.8 (26%); ^13^C NMR (CDCl_3_) 13.92 (s, CH_3_CH_2_CH_2_), 16.6 (d, *J* = 5.9 Hz, CH_3_CH_2_O), 20.3 (s, CH_3_CH_2_CH_2_), 31.83 (s, CH_2_CH_2_NH), 47.8 (d, *J* = 14.0 Hz, CH_2_N), 61.6 (d, *J* = 6.9 Hz, CH_2_O), 63.91 (d, *J* = 106.2 Hz, CHPh), 127.5 (d, *J* = 3.5 Hz, δ)^c^, 127.9 (d, *J* = 12.4 Hz, β′), 128.11 (s, γ′), 128.4 (d, *J* = 15.0 Hz, α′), 128.6 (d, *J* = 5.4 Hz, β)^d^, 132.1 (d, *J* = 2.8 Hz, δ′)^c^, 132.6 (d, *J* = 9.1 Hz, γ)^d^, ^c,d^ may be reversed.

For the diastereoisomers **6a-1** and **6a-2**: ^1^H NMR (CDCl_3_) δ 0.80 (t, *J*= 7.5 Hz) and 0.84 (t, *J* = 7.5 Hz) 3H, (CH_2_)_3_CH_3_ 1.20 (t, *J* = 7.0 Hz) and 1.204 (t, *J* = 7.0 Hz) 3H, OCH_2_CH_3_, 1.22–1.47 (m, 4H, (CH_2_)_2_, 2.33–2.56 (m, 2H, NCH_2_), 3.89–4.04 (m) and 4.15–4.22 (m, OCH_2_) partially overlapped by 4.10 (d, *J* = 15.0 Hz) and 4.17 (d, *J* = 15.0 Hz) PhCH, total intensity 3H, 7.11–7.68 (m, 10H, Ar); M + H = 332; [M + H]^+^_found_ = 332.1778, C_19_H_27_NO_2_P requires 332.1779.

**6b-1**: ^31^P NMR (CDCl_3_) δ 38.2 (66%); ^13^C NMR (CDCl_3_) 11.5 (s, CH_3_CH_2_CH_2_), 16.4 (d, *J* = 5.9 Hz, CH_3_CH_2_O), 22.8 (s, CH_3_CH_2_CH_2_), 49.8 (d, *J* = 14.7 Hz, CH_2_N), 61.5 (d, *J* = 7.1 Hz, CH_2_O), 63.84 (d, *J* = 107.7 Hz, CHPh), 127.6 (d, *J* = 3.1 Hz, δ)^a^, 128.04 (d, *J* = 12.2 Hz, β′), 128.10 (s, γ′), 128.8 (d, *J* = 5.4 Hz, β)^b^, 129.4 (d, *J* = 126.3 Hz, α), 132.2 (d, *J* = 2.7 Hz, δ′)^a^, 132.4 (d, *J* = 9.2 Hz, γ)^b^, 135.7 (d, *J* = 4.1 Hz, α′), ^a,b^ may be reversed.

**6b-2**: ^31^P NMR (CDCl_3_) δ 39.8 (34%); ^13^C NMR (CDCl_3_) 11.6 (s, CH_3_CH_2_CH_2_), 16.5 (d, *J* = 6.1 Hz, CH_3_CH_2_O), 22.9 (s, CH_3_CH_2_CH_2_), 50.0 (d, *J* = 14.2 Hz, CH_2_N), 61.6 (d, *J* = 6.8 Hz, CH_2_O), 63.85 (d, *J* = 107.1 Hz, CHPh), 127.5 (d, *J* = 3.4 Hz, δ)^c^, 127.88 (d, *J* = 12.3 Hz, β′), 128.01 (s, γ′), 128.6 (d, *J* = 5.4 Hz, β)^d^, 132.1 (d, *J* = 2.8 Hz, δ′)^c^, 132.6 (d, *J* = 9.1 Hz, γ)^d^, ^c,d^ may be reversed.

For the diastereoisomers **6b-1** and **6b-2**: ^1^H NMR (CDCl_3_) δ 0.8 (t, *J* = 7.4 Hz) and 0.85 (t, *J* = 7.4 Hz) 3H, (CH_2_)_3_CH_3_, 1.21 (t, *J* = 7.1 Hz) and 1.34 (t, *J* = 7.0 Hz) 3H, OCH_2_CH_3_, 1.37–1.54 (m, 2H, CH_2_), 2.34–2.56 (m, 2H, NCH_2_), 3.89–4.06 (m) and 4.16–4.25 (m, OCH_2_) partially overlapped by 4.12 (d, *J* = 14.8 Hz) and 4.20 (d, *J* = 16.9 Hz) PhCH, total intensity 3H, 7.13–7.68 (m, 10H, Ar); M + H = 318; [M + H]^+^_found_ = 318.1626, C18H25NO2P requires 318.1623.

**6c-1**: ^31^P NMR (CDCl_3_) 39.7 (51%), ^13^C (CDCl_3_) 16.4 (d, *J* = 6.0 Hz, CH_3_CH_2_O), 51.0 (d, *J* = 15.9 Hz, CH_2_N)^a^, 61.5 (d, *J* = 7.0 Hz, CH_2_O)^b^, 62.0 (d, *J* = 104.4 Hz, CHPh)^c^, 127.04 (s, δ″), 127.6 (d, *J* = 3.2 Hz, δ)^d^, 127.9 (d, *J* = 12.4 Hz, β′), 128.18 (s, β″), 128.30 (s, γ″), 128.33 (s, γ′), 128.8 (d, *J* = 5.4 Hz, β)^e^, 129.6 (d, *J* = 126.9 Hz, α), 132.21 (d, *J* = 2.8 Hz, δ′)^d^, 132.7 (d, *J* = 9.1 Hz, γ)^e^, 135.32 (bs, α′), 139.4 (s, α″).

**6c-2**: ^31^P NMR (CDCl_3_) 38.1 (49%), ^13^C (CDCl_3_) 16.6 (d, *J* = 6.0 CH_3_CH_2_O), 51.4 (d, *J* = 15.1 Hz, CH_2_N)^a^, 61.6 (d, *J* = 11.9 Hz, CH_2_O)^b^, 62.7 (d, *J* = 107.3 CHPh)^c^,126.97 (s, δ″), 127.8 (d, *J* = 2.9 Hz, δ)^d^, 128.5 (d, *J* = 12.8 Hz, β′), 128.18 (s, β″), 128.27 (s, γ′), 128.30 (s, γ″), 129.0 (d, *J* = 5.4 Hz, β)^e^, 129.6 (d, *J* = 126.9 Hz, α), 132.17 (d, *J* = 2.8 Hz, δ′)^d^, 132.4 (d, *J* = 9.2 Hz, γ)^e^, 135.35 (bs, α′), 139.2 (s, α″).

^a,b,c,d,e^ may be reversed/tentative assignments.

For the diastereoisomers **6c-1** and **6c-2**: ^1^H NMR (CDCl_3_) δ 1.19 (t, *J* = 7.5 Hz) and 1.34 (t, *J* = 7.5 Hz) 3H, OCH_2_CH_3_ 3.52 (d, *J* = 15.0 Hz) and 3.59 (d, *J* = 15.0 Hz) 1H, NCH, 3.82–4.25 (m, NCH + OCH_2_) partially overlapped by 4.07 (d, *J* = 15.0 Hz) and 4.18 (d, *J* = 15.0 Hz) PhCH, total intensity 4H, 7.07–7.71 (m, 15H, Ar); M + H = 366; [M + H]^+^_found_ = 366.1618, C_22_H_25_NO_2_P requires 366.1623.

**6d-1**: ^31^P NMR (CDCl_3_) δ 38.18 (62%); ^13^C NMR (CDCl_3_) δ 13.85 (s, CH_3_CH_2_CH_2_), 16.4 (d, *J* = 5.9 Hz, CH_3_CH_2_O), 20.1 (s, CH_3_CH_2_CH_2_), 21.10 (s, C_6_H_4_CH_3_), 31.8 (s, CH_2_CH_2_NH), 47.6 (d, *J* = 15.0 Hz, CH_2_N), 61.38 (d, *J* = 7.7 Hz, CH_2_O), 63.5 (d, *J* = 108.6 Hz, CHPh), 128.0 (d, *J* = 12.3 Hz, β′), 128.6 (d, *J* = 5.4 Hz, β)^a^, 128.8 (d, *J* = 2.5 Hz, γ′), 129.6 (d, *J* = 125.6 Hz, α), 132.1 (d, *J* = 2.8 Hz, δ), 132.4 (d, *J* = 9.3 Hz, γ) ^a^, 137.2 (d, *J* = 3.2 Hz, α′), ^a^ may be reversed.

**6d-2**: ^31^P NMR (CDCl3) δ 38.18 (38%); ^13^C NMR (CDCl_3_) δ 13.91 (s, CH_3_CH_2_CH_2_), 16.5 (d, *J* = 5.9 Hz, CH_3_CH_2_O), 20.2 (s, CH_3_CH_2_CH_2_), 21.06 (s, PHCH_3_), 31.9 (s, CH_2_CH_2_NH), 47.7 (d, *J* = 14.4 Hz, CH_2_N), 61.44 (d, *J* = 7.9 Hz, CH_2_O), 63.6 (d, *J* = 107.6 Hz, CHPh), 127.8 (d, *J* = 12.3 Hz, β′), 128.4 (d, *J* = 5.5 Hz, β)^b^, 128.7 (d, *J* = 2.8 Hz, γ′), 128.6 (d, *J* = 124.8 Hz, α), 132.0 (d, *J* = 2.7 Hz, δ′), 132.6 (d, *J* = 8.8 Hz, γ) ^b^, 137.0 (d, *J* = 3.5 Hz, α′), ^b^ may be reversed.

For the diastereoisomers **6d-1** and **6d-2**: ^1^H NMR (CDCl_3_) δ 0.73 (t, *J* = 7.5 Hz) and 0.76 (t, *J* = 7.5 Hz) 3H, (CH_2_)_3_CH_3_, 1.15 (t, *J* = 7.5) and 1.26 (t, *J* = 7.5 Hz) OCH_2_CH_3_, overlapped by 1.06–1.45 (m, (CH_2_)_2_), total intensity 7H, 2.22 (d, *J* = 5.0 Hz) and 2.26 (d, *J* = 5.0 Hz), 3H, C_6_H_4_CH_3_, 2.27–2.56 (m, 2H, NCH_2_), 4.01 (d, *J* = 15.0 Hz) and 4.13 (d, *J* ~ 15 Hz) PhCH, partially overlapped by 3.83–4.17 (m, OCH_2_), total intensity 3H, 6.90–7.62 (m, 9H, Ar); M + H = 346; [M + H]^+^_found_ = 346.1937, C_20_H_29_NO_2_P requires 346.1936.

**6e-1**: ^31^P NMR (CDCl_3_) δ 37.7 (56%); ^13^C NMR (CDCl_3_) 13.85 (s, CH_3_CH_2_CH_2_), 16.4 (d, *J* = 6.0 Hz, CH_3_CH_2_O), 20.1 (s, CH_3_CH_2_CH_2_), 31.7 (s, CH_2_CH_2_NH), 47.7 (d, *J* = 14.5 Hz, CH_2_NH), 61.6 (d, *J* = 7.0 Hz, CH_2_O), 63.4 (d, *J* = 107.2 Hz, CHPh), 128.13 (d, *J* = 6.2 Hz, β′)^a^, 128.24 (s, γ′), 128.0 (d, *J* = 125.6 Hz, α), 130.1 (d, *J* = 5.4 Hz, β)^b^, 132.4 (d, *J* = 9.0 Hz, γ)^b^, 133.4 (d, *J* = 3.8 Hz, δ), 134.3 (bs, α′).

**6e-2**: ^31^P NMR (CDCl_3_) 39.3 (43%), ^13^C (CDCl_3_) 13.89 (s, CH_3_CH_2_CH_2_), 16.5 (d, *J* = 6.1, CH_3_CH_2_O), 20.2 (s, CH_3_CH_2_CH_2_), 31.8 (s, CH_2_CH_2_NH), 47.8 (d, *J* = 14,1 Hz, CH_2_NH), 61.7 (d, *J* = 7.0 Hz, CH_2_O), 63.39 (d, *J* = 105.5 Hz, CHPh), 128.16 (d, *J* = 7.1 Hz, β′)^a^, 128.27 (s, γ′), 129.9 (d, *J* = 5.4 Hz, β)^c^, 132.6 (d, *J* = 9.1 Hz, γ)^c^, 133.2 (d, *J* = 3.9 Hz, δ), 134.2 (bs, α′).

^a,b,c^ may be reversed.

For the diastereoisomers **6e-1** and **6e-2**: ^1^H NMR (CDCl_3_) δ 0.84 (t, *J* = 7.3 Hz) and 0.85 (t, *J* = 7.3 Hz) 3H, (CH_2_)_3_CH_3_, 1.25 (t, *J* = 7.1 Hz) and 1.35 (t, *J* = 7.1 Hz) OCH_2_CH_3_ overlapped by 1.17–1.53 (m, (CH_2_)_2_) total intensity 7H, 2.32–2.60 (m, NCH_2_, 2H), 3.94–4.28 (m, OCH_2_) partially overlapped by 4.14 (d, *J* = 14.7 Hz) and 4.21 (d, *J* ~ 15 Hz) ArCH, total intensity 3H, 7.1–7.67 (m, 9H, Ar); M + H = 366; [M + H]^+^_found_ = 366.1380, C_19_H_26_ClNO_2_P requires 366.1390 for the ^35^Cl isotope.

**6f-1**: ^31^P NMR (CDCl_3_) δ 39.7 (52%); ^13^C NMR (CDCl_3_) 13.62 (s, CH_3_CH_2_CH_2_), 13.91 (s, CH_3_CH_2_CH_2_), 18.8 (s, CH_3_CH_2_CH_2_), 20.2 (s, CH_3_CH_2_CH_2_), 31.9 (s, CH_2_CH_2_NH), 32.6 (d, *J* = 6.3 Hz, CH_2_CH_2_O), 47.9 (d, *J* = 13.9 Hz, CH_2_N), 64.0 (d, *J* = 107.1 Hz, CHPh), 65.2 (d, *J* = 7.6 Hz, CH_2_O), 127.4 (b, δ)^a^, 127.8 (d, *J* = 13.9 Hz, β′), 127.9 (s, γ′), 128.8 (d, *J* = 3.8 Hz, β)^b^, 129.4 (d, *J* = 126.0 Hz, α), 132.1 (b, δ′)^a^, 132.7 (d, *J* = 8.8 Hz, γ)^b^.

**6f-2**: ^31^P NMR (CDCl_3_) δ 38.0 (48%); ^13^C NMR (CDCl_3_) 13.55 (s, CH_3_CH_2_CH_2_), 13.86 (s, CH_3_CH_2_CH_2_), 18.7 (s, CH_3_CH_2_CH_2_), 20.1 (s, CH_3_CH_2_CH_2_), 31.8 (s, CH_2_CH_2_NH), 32.5 (d, *J* = 6.3 Hz, CH_2_CH_2_O), 47.6 (d, *J* = 15.1 Hz, CH_2_N), 63.9 (d, *J* = 108.4 Hz, CHPh), 65.0 (d, *J* = 6.3 Hz, CH_2_O), 127.6 (b, δ)^a^, 128.05 (d, *J* = 12.6 Hz, β′), 128.06 (s, γ′), 128.6 (d, *J* = 5.0 Hz, β)^c^, 132.15 (b, δ′)^a^, 132.4 (d, *J* = 8.8 Hz, γ)^c^.

^a,b,c^ may be reversed.

For the diastereoisomers **6f-1** and **6f-2**: ^1^H NMR (CDCl_3_) δ 0.82 (t, *J* = 7.3 Hz) and 0.93 (t, *J* = 7.3 Hz) 3H, (CH_2_)_3_CH_3_, 0.860 (t, *J* = 7.3 Hz) and 0.865 (t, *J* = 7.3 Hz) 3H, (CH_2_)_3_CH_3_, 1.18–1.75 (m, 8H, (CH_2_)_2_, (CH_2_)_2_), 2.39–2.66 (m, 2H, NCH_2_), 3.83–4.20 (m, OCH_2_), partially overlapped by 4.15 (d, *J* = 16.9 Hz) and 4.26 (d, *J* = 16.9 Hz) PhCH, total intensity 3H, 7.10–7.74 (m, 10H, Ar); M + H = 360; [M + H]^+^_found_ = 360.2094, C_21_H_31_NO_2_P requires 360.2092.

**6g-1**: ^31^P NMR (CDCl_3_) δ 37.9 (51%); ^13^C NMR (CDCl_3_) 11.5 (s, CH_3_CH_2_CH_2_CH_2_), 13.5 (s, CH_3_CH_2_CH_2_), 18.7 (s, CH_3_CH_2_CH_2_CH_2_), 22.8 (s, CH_2_CH_2_NH), 32.5 (d, *J* = 6.0 Hz, CH_2_CH_2_O), 49.8 (d, *J* = 14.8 Hz, CH_2_NH), 63.8 (d, *J* = 107.8 Hz, CHPh), 65.0 (d, *J* = 7.2 Hz, CH_2_O), 127.6 (d, *J* = 3.1 δ)^a^, 127.8 (d, *J* = 12.5 Hz, β′), 128.05 (d, *J* = 2.5 Hz, γ′)^b^, 128.8 (d, *J* = 5.4 Hz, β), 129.4 (d, *J* = 126.0 Hz, α), 132.2 (d, *J* = 2.8, δ′)^a^, 132.4 (d, *J* = 9.0 Hz, γ)^b^, 135.77 (s, α′).

**6g-2**: ^31^P NMR (CDCl_3_) δ 39.7 (49%); ^13^C NMR (CDCl_3_) 11.6 (s, CH_3_CH_2_CH_2_CH_2_), 13.6 (s, CH_3_CH_2_CH_2_), 18.8 (s, CH_3_CH_2_CH_2_CH_2_), 22.9 (s, CH_2_CH_2_NH), 32.6 (d, *J* = 6.0 Hz, CH_2_CH_2_O), 50.0 (d, *J* = 14.2 Hz, CH_2_NH), 63.9 (d, *J* = 106.9 Hz, CHPh), 65.1 (d, *J* = 7.1 Hz, CH_2_O), 127.4 (d, *J* = 3.3 δ)^c^, 128.04 (d, *J* = 12.4 Hz, β′), 127.9 (d, *J* = 2.7 Hz, γ′)^d^, 128.6 (d, *J* = 5.4 Hz, β), 132.1 (d, *J* = 2.8, δ′)^c^, 132.7 (d, *J* = 9.0 Hz, γ)^d^, 135.80 (s, α′).

^a,b,c,d^ may be reversed.

For the diastereoisomers **6g-1** and **6g-2**: ^1^H NMR (CDCl_3_) δ 0.81 (t, *J* = 7.4 Hz) and 0.93 (t, *J* = 7.4 Hz) 3H, (CH_2_)_3_CH_3_, 0.860 (t, *J* = 7.4 Hz) and 0.865 (t, *J* = 7.4 Hz) 3H, (CH_2_)_2_CH_3_, 1.25–1.75 (m, 6H, (CH_2_)_2_, CH_2_), 2.36–2.61 (m, 2H, NCH_2_), 3.82–4.20 (m, OCH_2_) partially overlapped by 4.16 (d, *J* = 169 Hz) and 4.26 (d, *J* = 16.9 Hz) PhCH, total intensity 3H, 7.15–7.75 (m, 10H, Ar); M + H = 346; [M + H]^+^_found_ = 346.1943, C_20_H_29_NO_2_P requires 346.1936.

**6h-1:** ^31^P NMR (CDCl_3_) δ 37.9 (65%); ^13^C NMR (CDCl_3_) 13.6 (s, CH_3_CH_2_CH_2_), 18.7 (s, CH_3_CH_2_CH_2_), 32.5 (d, *J* = 5.9 Hz, CH_2_CH_2_O), 50.9 (d, *J* = 15.9 Hz, CH_2_NH), 61.4 (d, *J* = 109.8 Hz, CHPh), 65.0 (d, *J* = 7.0 Hz, CH_2_O), 126.97 (s, δ″), 127.8 (d, *J* = 2.9 Hz, δ)^a^, 128.1 (d, *J* = 12.8 Hz, β′), 129.0 (bs, β), 129.5 (d, *J* = 126.5 Hz, α), 132.21 (d, *J* = 3.1, δ′)^a^, 132.5 (d, *J* = 9.4 Hz, γ), 135.3 (bs, α′) 139.2 (s, α″), ^a^ may be reversed.

**6h-2:** ^31^P NMR (CDCl3) δ 39.6 (35%); ^13^C NMR (CDCl_3_) 13.7 (s, CH_3_CH_2_CH_2_), 18.9 (s, CH_3_CH_2_CH_2_), 32.7 (d, *J* = 6.2 Hz, CH_2_CH_2_O), 51.3 (d, *J* = 15.0 Hz, CH_2_NH), 62.6 (d, *J* = 107.5 Hz, CHPh), 65.2 (d, *J* = 7.1 Hz, CH_2_O), 127.04 (s, δ″), 127.6 (d, *J* = 3.2 Hz, δ)^b^, 127.9 (d, *J* = 12.6 Hz, β′), 128.8 (s, β), 132.19 (d, *J* = 3.4, δ′) ^b^, 132.7 (d, *J* = 9.1 Hz, γ), 135.4 (bs, α′) 139.3 (s, α″), ^b^ may be reversed.

For the diastereoisomers **6h-1** and **6h-2**: ^1^H NMR (CDCl_3_) δ 0.84 (t, *J* = 7.4 Hz) and 0.93 (t, *J* = 7.4 Hz) 3H, (CH_2_)_3_CH_3_, 1.21–1.72 (m, 4H, (CH_2_)_2_), 3.51–3.64 (m, 1H, NCH), 3.80–3.97 + 4.11–4.17 (m, 3H, NCH + OCH_2_), 4.06 (d, *J* = 15.5 Hz) and 4.20 (d, *J* = 16.9 Hz) 1H, PhCH, 7.05–7.75 (m, 15H, Ar); M + H = 394; [M + H]^+^_found_ = 394.1932, C_24_H_29_NO_2_P requires 394.1936.

#### 3.3.2. General Procedure for the Synthesis of Alkoxy α-Alkylamino-benzyl-phenylphosphinates **6a** and **6f** by the Kabachnik–Fields Condensation

A mixture of 0.20 mL (2.0 mmol) of benzaldehyde, 0.20 mL (2.0 mmol) of butylamine and 2.4 mmol of phosphinate [ethyl-phenyl-*H*-phosphinate: 0.36 mL, butyl-phenyl-*H*-phosphinate: 0.48 g] was added to the MW glass vessel. Then, the resulting mixture was irradiated in a CEM microwave reactor equipped with a pressure-controller at 135 °C for 1.5–2 h. The residue so obtained was purified by column chromatography (silica gel, acetone:hexane 8:2) to afford α-aminophophonates **6a** (0.36 g, 54%) and **6f** (0.52 g, 75%) as oils.

### 3.4. Bioactivity Studies

This study utilized two cell lines: human myeloma (U266) and human pancreatic ductal adenocarcinoma (PANC-1), both sourced from the European Collection of Authenticated Cell Cultures (ECACC, Salisbury, UK). The PANC-1 cell line exhibits adherent growth characteristics (87092802 ECACC), whereas U266 cells (85051003 ECACC) grow in suspension. PANC-1 cells were cultured in DMEM medium (Sigma Ltd., St. Louis, MO, USA), while U266 cells were maintained in RPMI 1640 medium (Sigma Ltd., St. Louis, MO, USA). Both media were supplemented with 10% fetal bovine serum (Invitrogen Corporation, New York, NY, USA), 1% L-glutamine (Invitrogen Corporation, New York, NY, USA), and 1% penicillin/streptomycin (Invitrogen Corporation, New York, NY, USA).

#### Cell Viability Assays

Tested compounds were dissolved in dimethyl sulfoxide (DMSO; AppliChem GmbH, Darmstadt, Germany) at a stock concentration of 10^−1^ M, ensuring the final DMSO concentration remained below 1% (*v*/*v*). Stock solutions were stored at −80 °C, and fresh solutions were prepared before each experiment.

The viability of PANC-1 cells following treatment was assessed using the xCELLigence system, a non-invasive impedimetric technique. Adherent PANC-1 cells were seeded at a density of 10^5^ cells/mL in 96-well plates (E-Plate 96 PET; ACEA Biosciences, San Diego, CA, USA) equipped with gold microelectrodes to detect impedance changes associated with cell proliferation. Baseline impedance readings were taken in a cell-free medium prior to seeding. After overnight incubation, cells were exposed to the compounds at final concentrations of 1, 10, and 100 μM, along with appropriate controls (medium and DMSO). Data were collected using RTCA 2.0 software (Real-Time Cell Analyzer; ACEA Biosciences, San Diego, CA, USA).

For U266 cells, which grow in suspension, the xCELLigence system was not suitable. Instead, cell viability was measured using the CellTiter-Glo Luminescent Cell Viability Assay (Promega, Madison, WI, USA). U266 cells were seeded in white-walled 96-well plates (Thermo Scientific, Waltham, MA, USA) at a density of 10^5^ cells/mL. After overnight incubation, cells were treated with the test compounds at 1, 10, and 100 μM concentrations, along with respective controls (medium and DMSO). Following a 72 h incubation period, CellTiter-Glo Reagent was added to each well, and luminescence was measured using a Fluoroskan FL Microplate Fluorometer and Luminometer (Thermo Scientific, Waltham, MA, USA).

All experiments were performed in triplicate, and the results were normalized to the untreated medium control, presented as mean ± standard deviation (SD).

## 4. Conclusions

In summary, three approaches for the synthesis of the commercially unavailable butyl phenyl-*H*-phosphinate, including alkylating esterification (**A**), MW-assisted (**B**) and thermal direct esterification (**C**) of phenyl-*H*-phosphinic acid, were investigated. From among the thermal variation, it seemed to be the most suitable regarding scalability, although the latter transformation required a prolonged reaction time. The side reactions regarding the formation of the pyrophosphinate in the MW-promoted version, and the formation of the phenylphosphonic acid butyl ester (by oxidation) in the thermal variation were also observed and possibly eliminated. In the second phase of our study, butyl phenyl-*H*-phosphinate and ethyl phenyl-*H*-phosphinate, the latter commercially available, were utilized in the synthesis of α-aminobenzyl-phenylphosphinates. From among the two possibilities offered by the aza-Pudovik addition and the three-component Kabachnik–Fields condensation, the first method seemed to be more clear-cut and more efficient. Hence, in the first step, the corresponding imines were prepared by the condensation of benzaldehyde derivatives and primary amines. This was followed by the MW-assisted addition of the alkyl phenyl-*H*-phosphinates on the C=N double bond of the imines. Altogether, eight new α-alkylaminophosphinates formed as the mixture of two diastereoisomers were synthesized and characterized. A few derivatives were subjected to testing for cytotoxic activity. Altogether, the aminophosphonic derivatives synthesized had only a slight impact on the viability of the two investigated (U266 and PANC-1) cell lines; however, the butyl (α-butylamino-benzyl-)phenylphosphinate was effective against PANC-1 cells at the highest investigated concentration, even lowering the cell viability to 30% compared to the control. On the other hand, ethyl (α-benzylamino-benzyl-)phenylphosphinate showed cytotoxic activity on U266 myeloma cells.

## Figures and Tables

**Table 1:** Effect of the size of the MW batch on the outcome of the esterification.

Entry	1 (mmol)	T (°C)	t (h)	Conversion (%)	Composition (%) ^[a]^		
					1	2	3
1	0.70	160	1.5	100	–	100 ^[b]^	–
2	1.40	160	2.5	86	14	77 ^[c]^	9
3	1.40	180	1.5	78	22	69	9
4	2.80	185	3.5	59	41	54	5
5	2.80	200	4	50	50	45	5

^[a]^ Determined on the basis of relative ^31^P NMR intensities. ^[b]^ Phosphinate **2** was isolated in an 80% yield after flash chromatography. ^[c]^ Phosphinate **2** was isolated in a 71% yield after flash chromatography.

**Table 2:** Scale-up experiments on the esterification of phenyl-*H*-phosphinic acid **1** with butyl alcohol on conventional heating (Δ means reflux).

Entry	1 (mmol)	BuOH (Equiv.)	T (°C)	t (h)	Conversion (%)	Composition (%) ^[a]^		
						1	2	4
1	1.4	15	Δ	36	98	2	97	1
2	14.1	12	Δ	48	95 ^[b]^	5	86	9
3	2.8	15	110 ^[c]^ (oil bath)	72	100		100	–
4	14.1	12	Δ ^[c]^	60	100 ^[d]^	0	100	–
5	3.52	12	Δ ^[c,e]^	30	100 ^[f]^	0	100	–

^[a]^ Determined on the basis of ^31^P NMR intensities. ^[b]^ Phosphinate **2** was isolated in a yield of 80%. ^[c]^ Under N_2_ atmosphere. ^[d]^ Phosphinate **2** was obtained in a yield of 95%. ^[e]^ In the presence of 10% of [bmim][PF_6_]. ^[f]^ Phosphinate **2** was obtained in a yield of 80%.

**Table 3:** Identification of the butyl phenyl-*H*-phosphinate **2** and by-products **3** and **4**.

	δ_P_ (CDCl_3_)	δ_P_ [Lit.]	[M + H]	HRMS
PhP(O)(OBu)H **2**	24.8	25.1 [36]	199	[M + H]_found_ = 190.0888, C_10_H_16_O_2_P requires 199.0888
PhHP(O)OP(O)PhH **3**	−0.90	–	267	
PhP(O)(OBu)OH **4**	20.9	–	215	

**Table 4:** The antiproliferative effects of the aminophosphinates **6a**–**f** (1, 10, and 100 μM) on PANC-1 and U266 cell lines were evaluated after 72 h. Data are presented as mean ± SD (*n* = 3). Statistical significance was assessed using a one-way ANOVA test followed by Fisher’s LSD post hoc test, with significance levels indicated as follows: x: *p* < 0.05, y: *p* < 0.01, z: *p* < 0.001.

		PANC-1			U266	
Compound	1 μM	10 μM	100 μM	1 μM	10 μM	100 μM
**6a**	0.98 ± 0.03	1.03 ± 0.12	1.20 ± 0.04 ^x^	1.03 ± 0.08	0.95 ± 0.07	0.94 ± 0.06
**6b**	0.95 ± 0.13	1.17 ± 0.19	0.95 ± 0.13	1.10 ± 0.05	1.15 ± 0.02 ^x^	0.94 ± 0.07
**6c**	1.04 ± 0.37	1.00 ± 0.24	1.08 ± 0.14	0.73 ± 0.25 ^z^	1.05 ± 0.12	1.06 ± 0.05 ^x^
**6d**	1.16 ± 0.11	0.97 ± 0.07	1.15 ± 0.12	1.09 ± 0.08	1.18 ± 0.08 ^x^	0.99 ± 0.02
**6e**	1.05 ± 0.27	1.07 ± 0.10	1.20 ± 0.17	1.13 ± 0.02 ^x^	1.16 ± 0.03 ^x^	0.97 ± 0.01
**6f**	0.96 ± 0.02	0.93 ± 0.12	0.30 ± 0.15 ^z^	1.06 ± 0.04	0.81 ± 0.09 ^y^	0.91 ± 0.02

## Data Availability

All experimental data and documentation are included in the paper and in the Supplementary Material.

## Supplementary Materials

The following supporting information can be downloaded at: https://www.mdpi.com/article/10.3390/molecules30020339/s1, Includes the ^31^P, ^13^C and ^1^H NMR, as well as the LC-MS spectra of products **6a**–**h**.
