# Supplementary material for: Synthesis of Alkyl α-Amino-benzylphosphinates by the Aza-Pudovik Reaction; The Preparation of the Butyl Phenyl-H-phosphinate Starting P-Reagent
Source: Molecules. 2025 Jan 16;30(2):339. doi: 10.3390/molecules30020339 (PMC11767357; doi:10.3390/molecules30020339)
Supplement: Supplementary file 1 [file molecules-30-00339-s001.zip › molecules-3383438-supplementary.pdf]

Supplementary Materials

Synthesis of Alkyl α-Amino-benzylphosphinates by the Aza-Pudovik Reaction; The Preparation of Butyl Phenyl-*H*-phosphinate Starting P-Reagent

Bence Bajusz [1], Dorka Nagy [1], Regina Tóth [1], Zsuzsanna Szalai [1], Ágnes Gömöry[2], Angéla Takács [3], László Kőhidai [3] and György Keglevich [1,*]

[1]Department of Organic Chemistry and Technology, Faculty of Chemical Technology and Biotechnology, Budapest University of Technology and Economics, Műegyetem rkp. 3, 1111 Budapest, Hungary; bajusz.bence@edu.bme.hu (B.B.); nagydorka0801@gmail.com (D.N.); regina@icont.hu (R.T); sz.zsuzsi97@gmail.com (Z.S.)

[2]MS Proteomics Research Group, Research Centre for Natural Sciences, 1117 Budapest, Hungary; gomory.agnes@ttk.hu

[3]Department of Genetics, Cell- and Immunobiology, Semmelweis University, Nagyvárad tér 4, 1089 Budapest, Hungary; takacs.angela@semmelweis.hu (A.T.); kohlasz2@gmail.com (L.K.)

**Correspondence: keglevich.gyorgy@vbk.bme.hu; Tel.: +36-1-463-1111 (ext. 5883)*

Table of contents

1. ^{31}P, ^{13}C, ^{1}H NMR spectra for the compounds **6a-h** synthesized....................................S2
2. HPLC-MS analysis for the compounds **6a-c,e,f,h** synthesized....................................S14

1. ^{31}P, ^{13}C, ^{1}H NMR spectra for the compounds 6a-h synthesized

^{31}P {^{1}H} NMR (202 MHz, $CDCl_3$) spectra for 6a

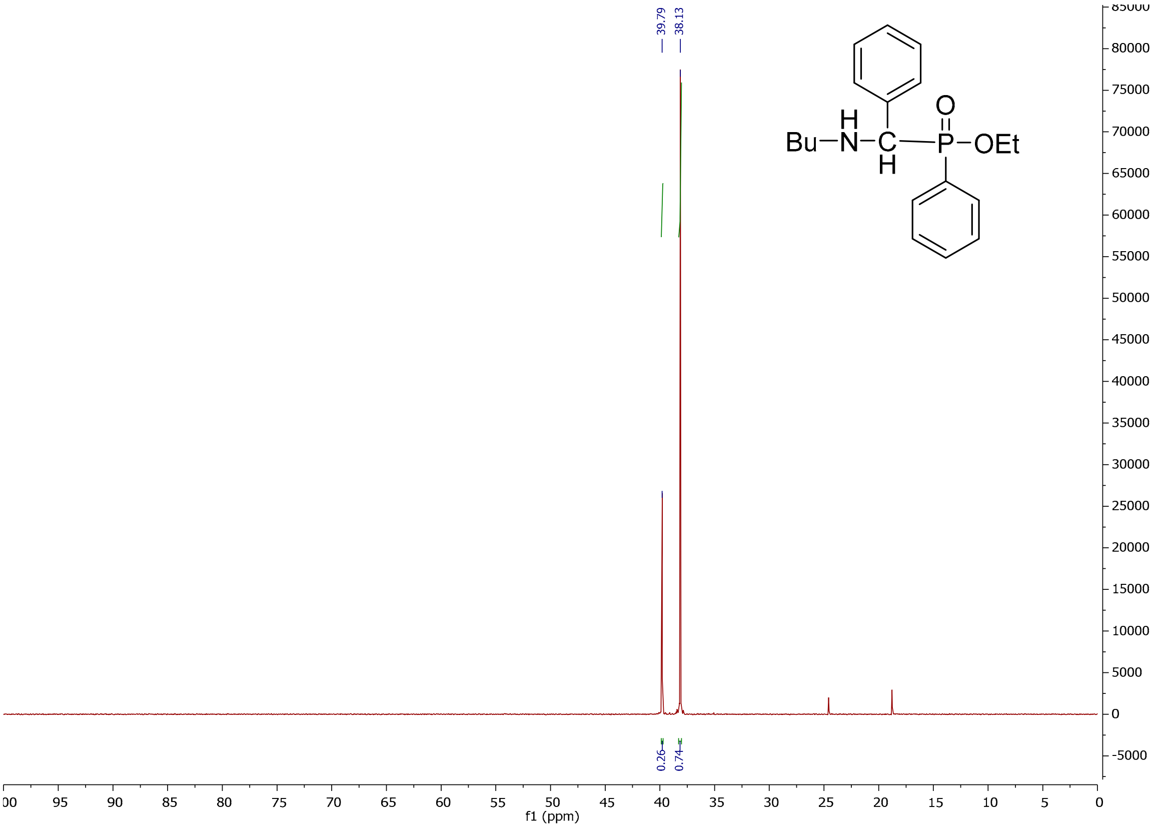

^{13}C {^{1}H} NMR (202 MHz, $CDCl_3$) spectra for 6a

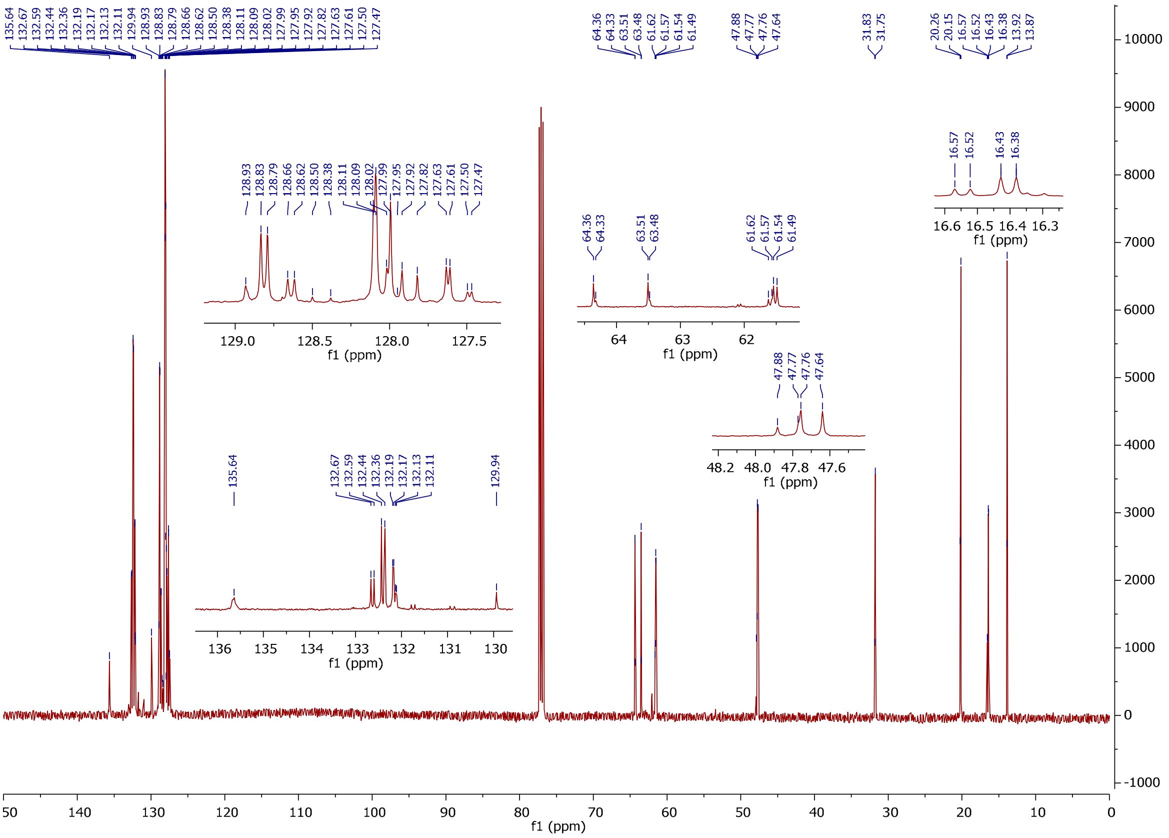

^{1}H NMR (500 MHz, CDCl$_3$) spectra for 6a

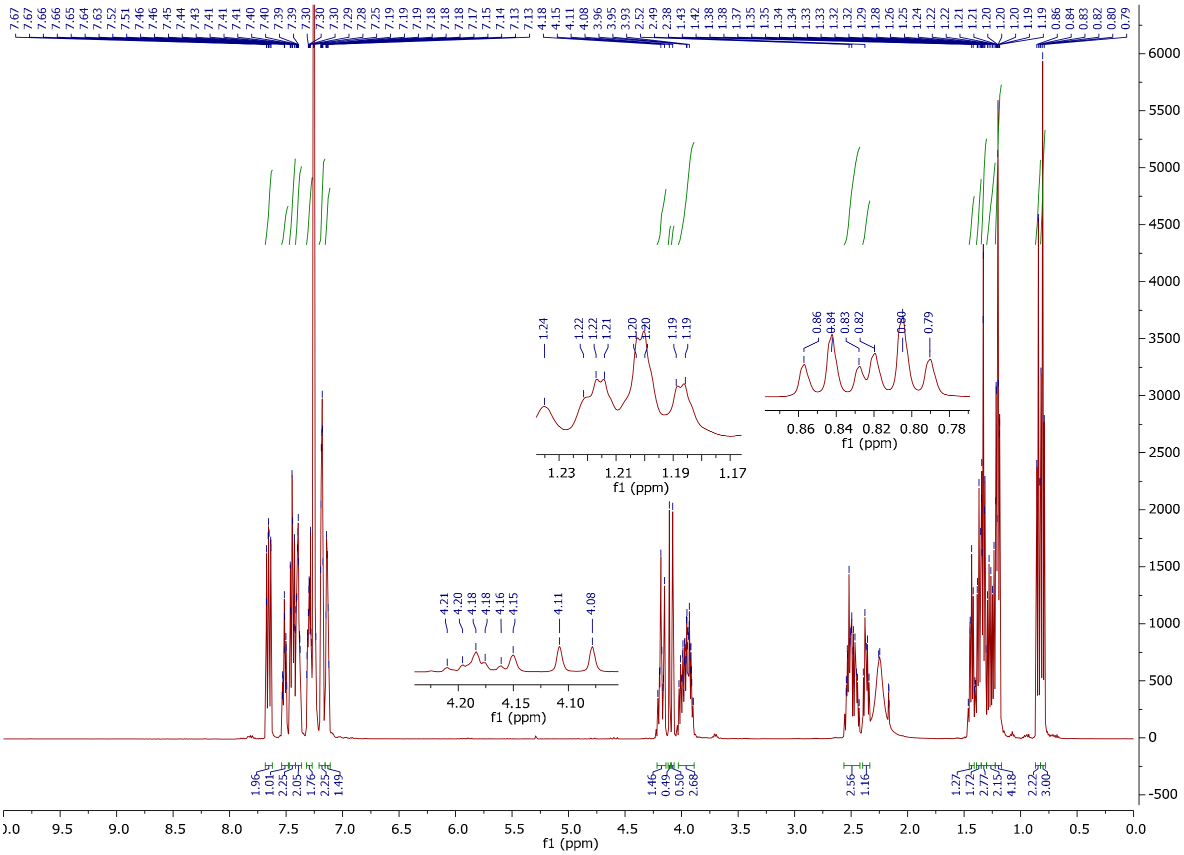

^{31}P {^{1}H} NMR (202 MHz, CDCl$_3$) spectra for 6b

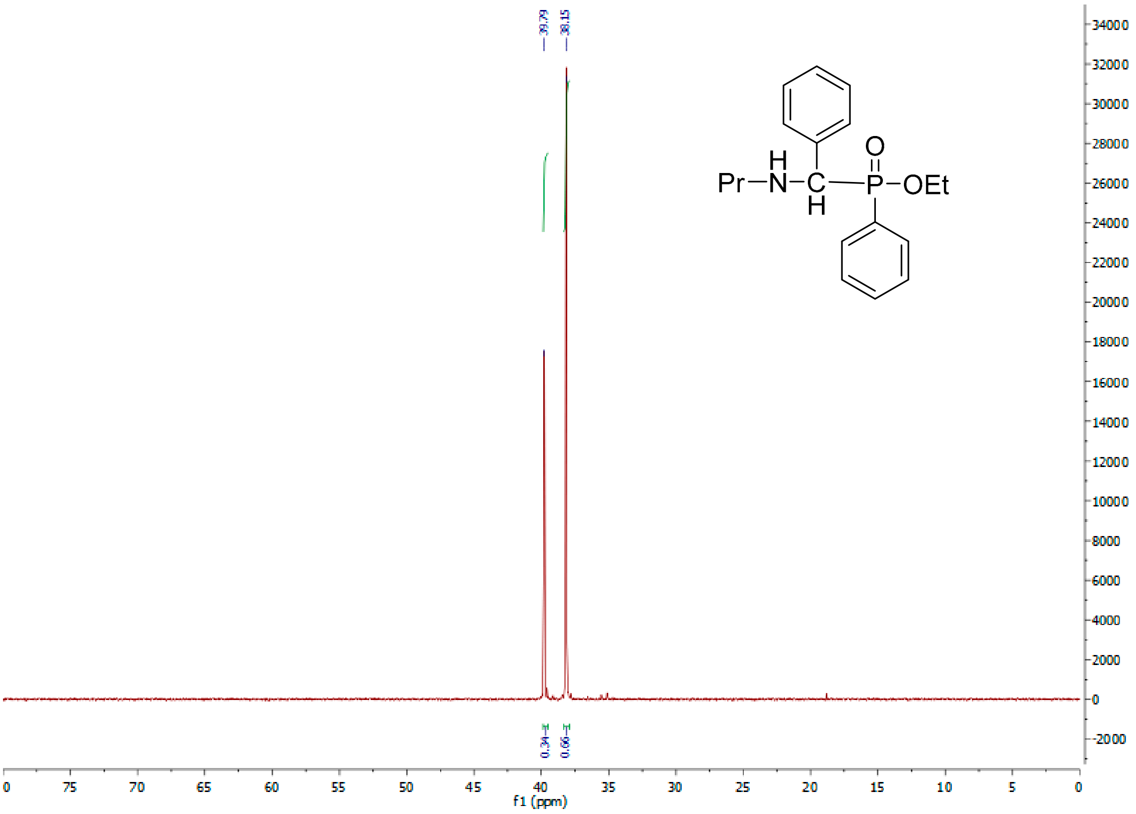

$^{13}C\{^{1}H\}$ NMR (202 MHz, $CDCl_3$) spectra for 6b

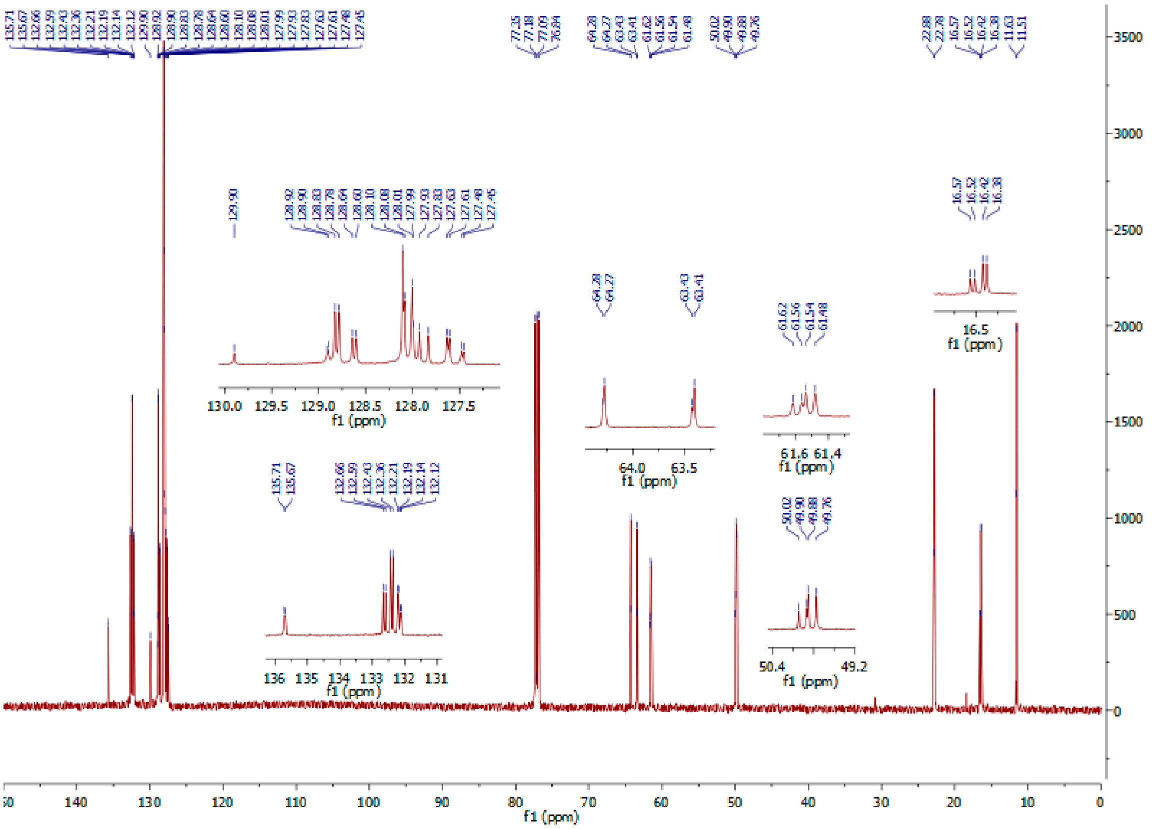

^{1}H NMR (500 MHz, $CDCl_3$) spectra for 6b

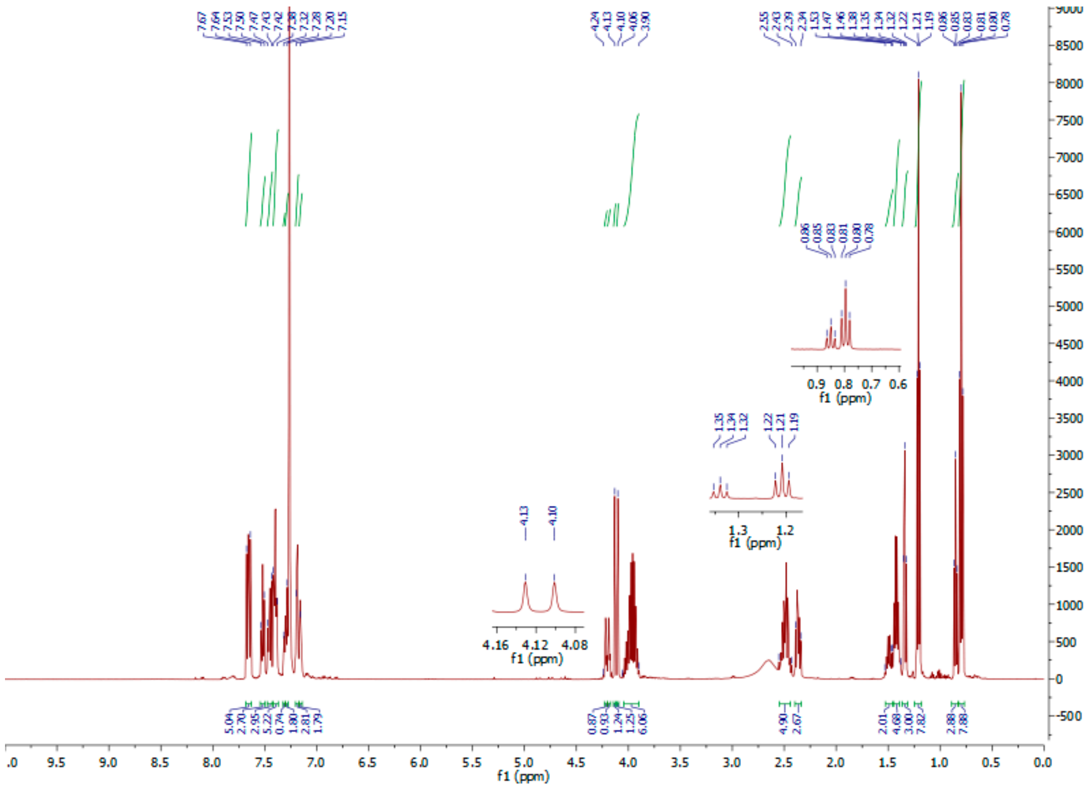

^{31}P {^{1}H} NMR (202 MHz, CDCl$_3$) spectra for 6c

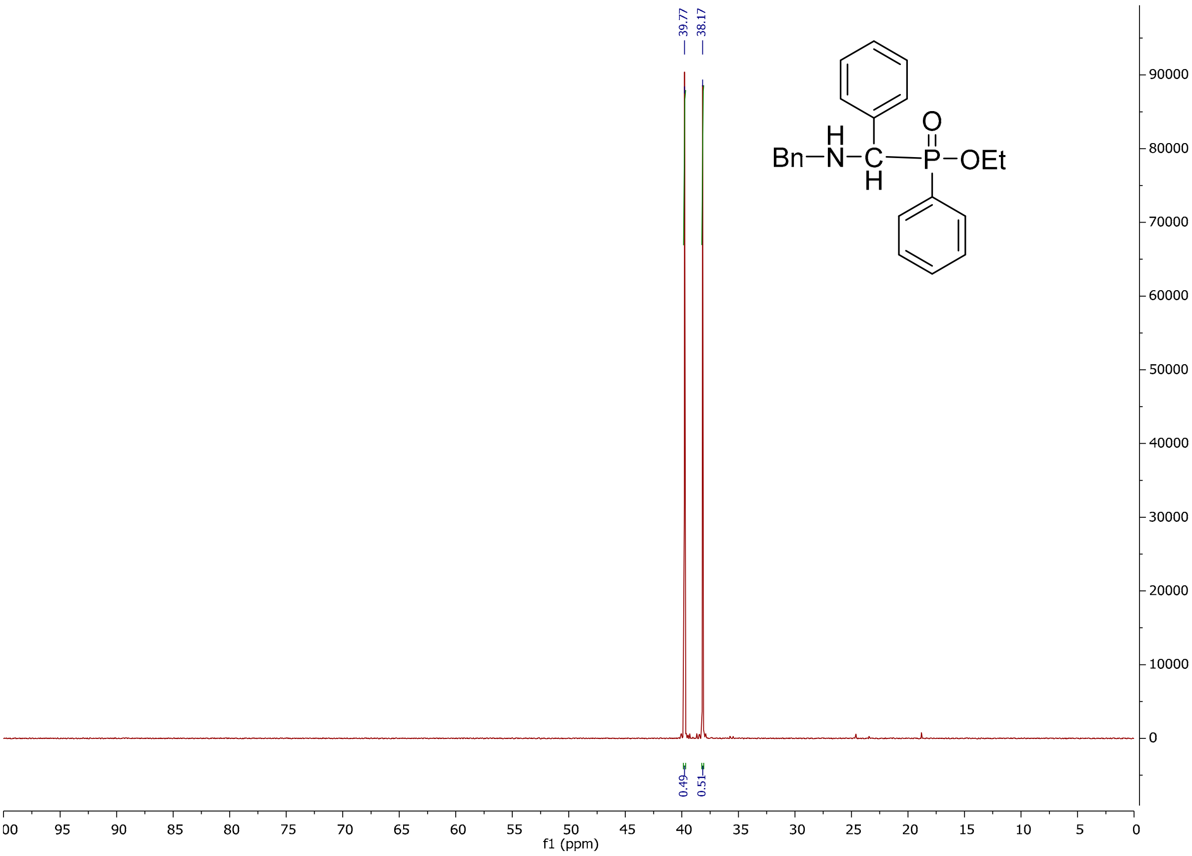

^{13}C {^{1}H} NMR (202 MHz, CDCl$_3$) spectra for 6c

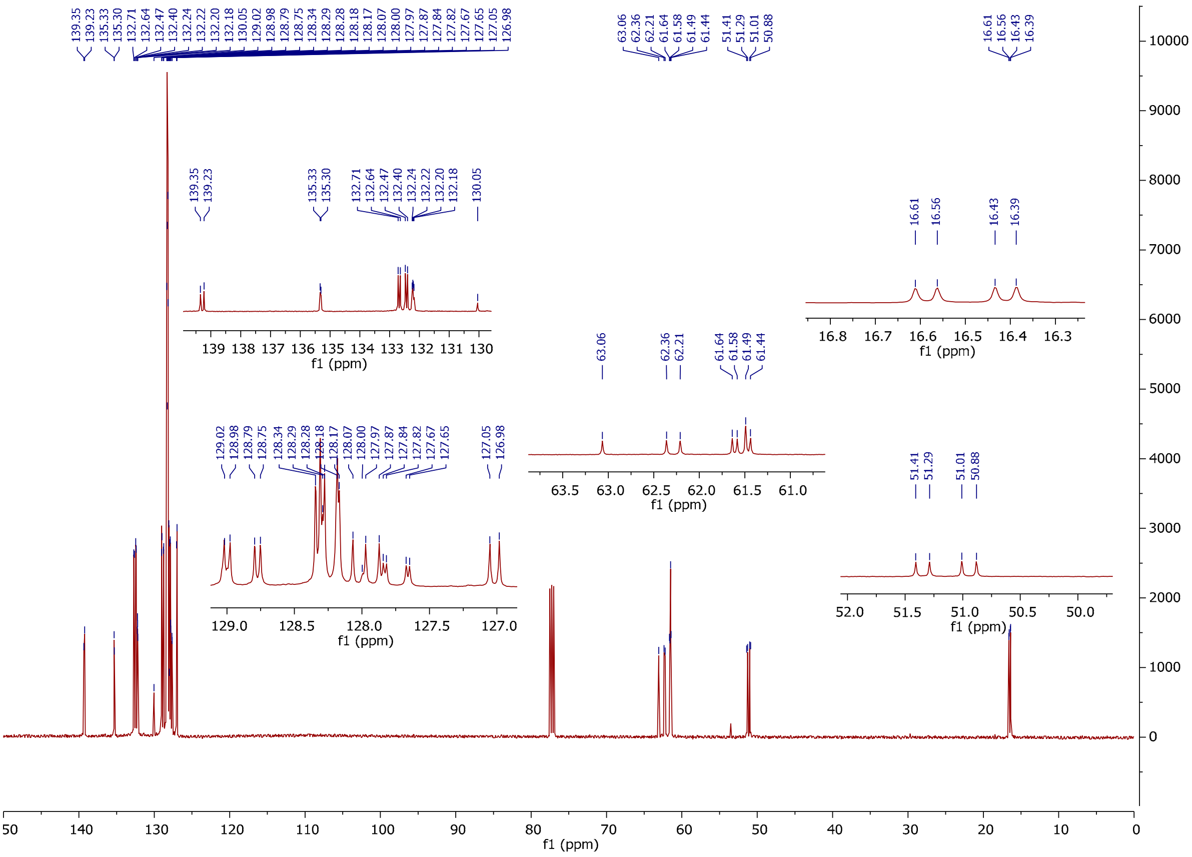

^{1}H NMR (500 MHz, CDCl$_3$) spectra for 6c

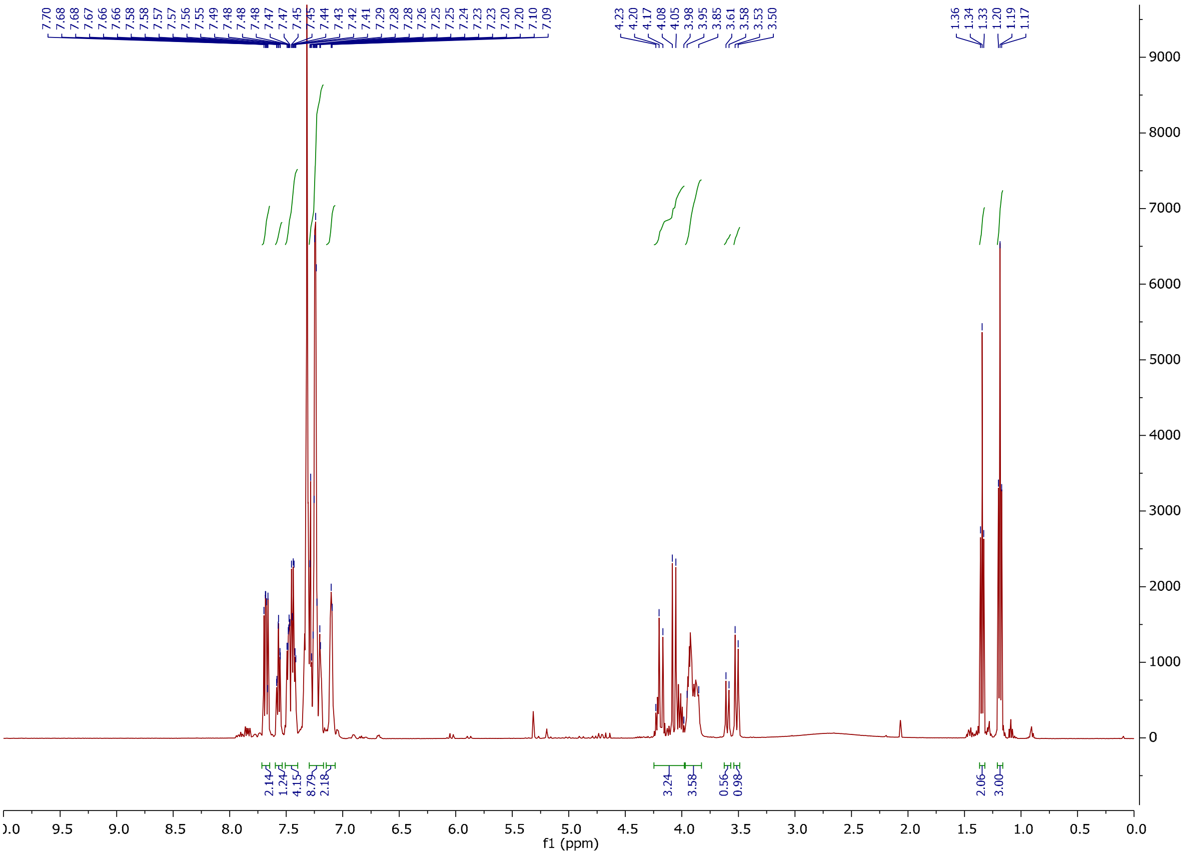

^{31}P {^{1}H} NMR (202 MHz, CDCl$_3$) spectra for 6d

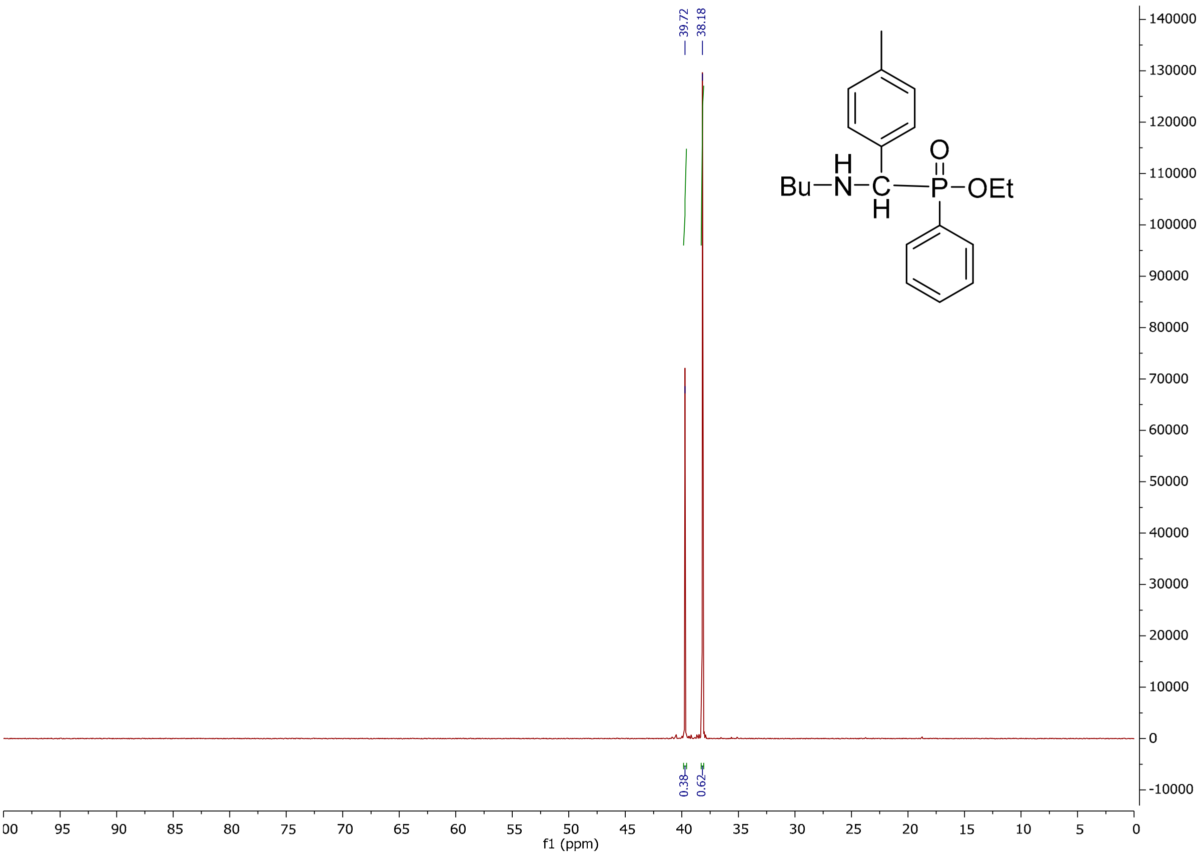

^{13}C {^{1}H} NMR (202 MHz, $CDCl_3$) spectra for 6d

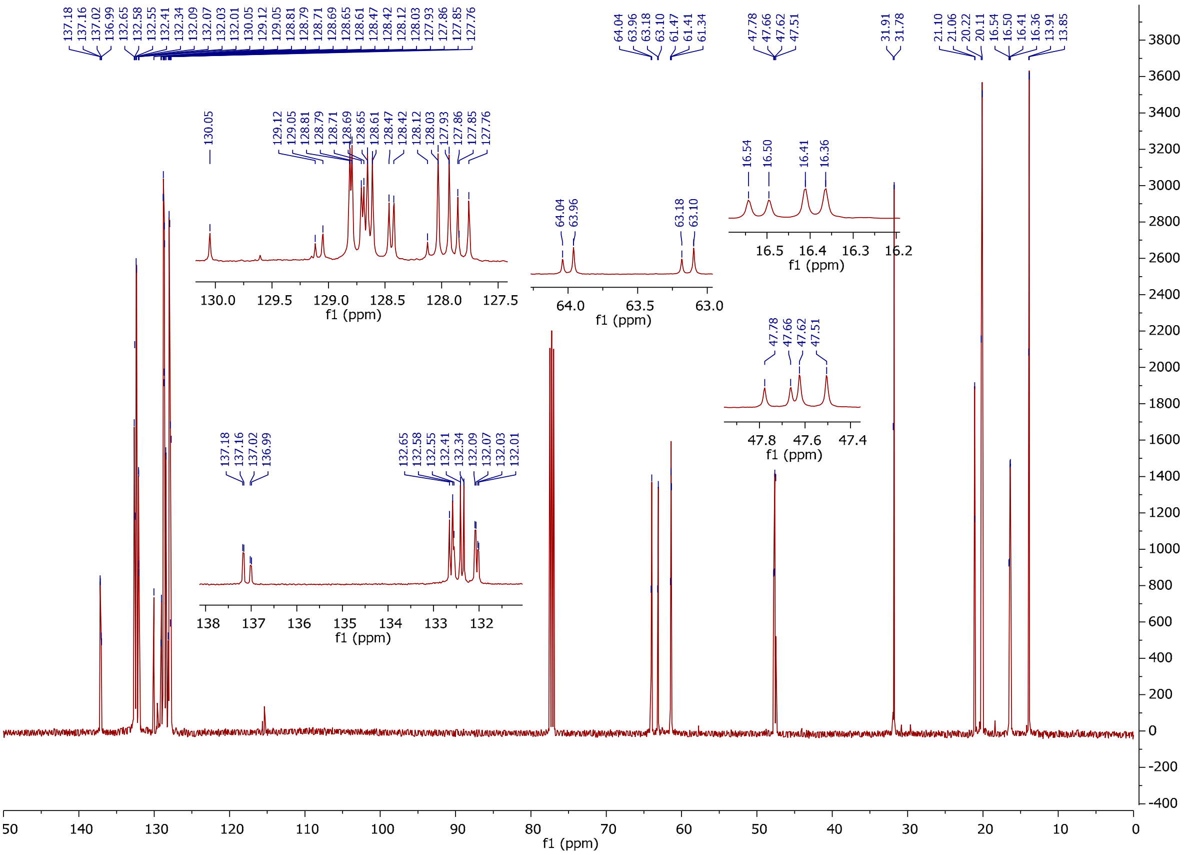

^{1}H NMR (500 MHz, $CDCl_3$) spectra for 6d

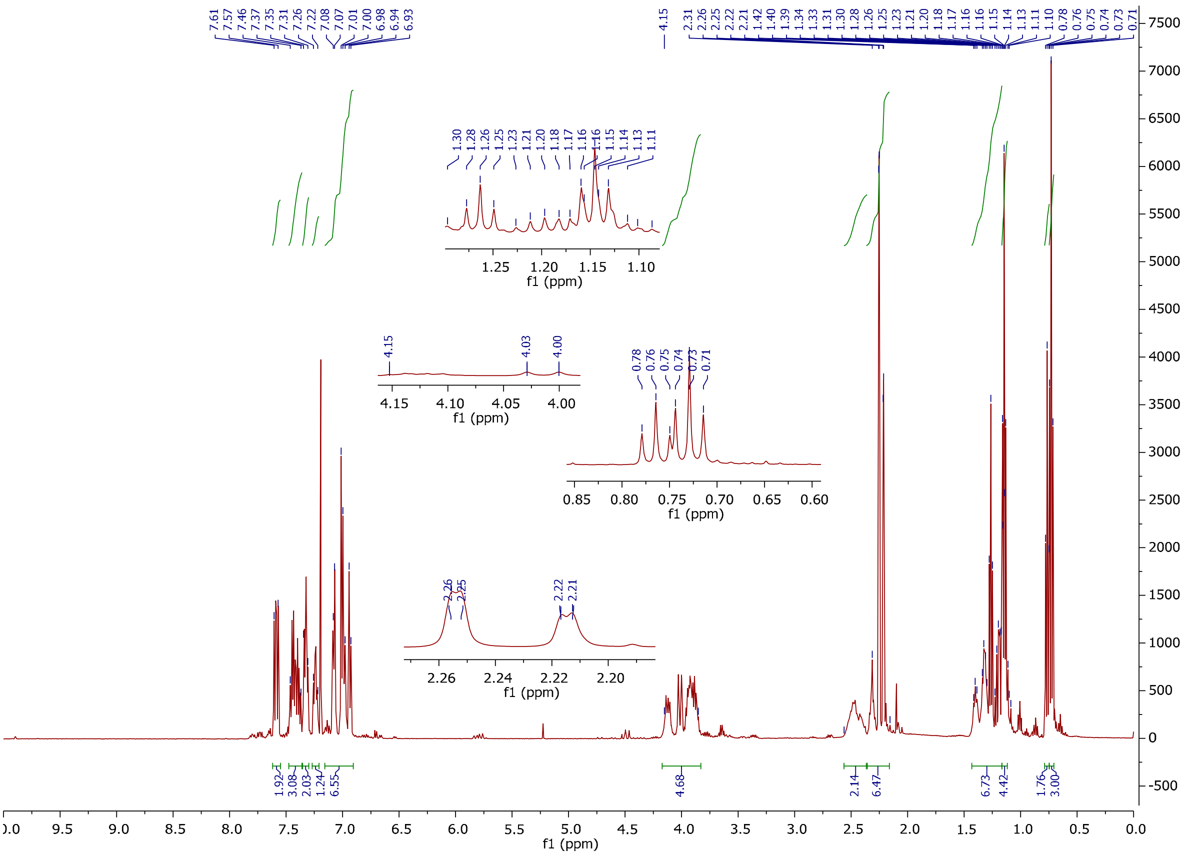

^{31}P {^{1}H} NMR (202 MHz, CDCl$_3$) spectra for 6e

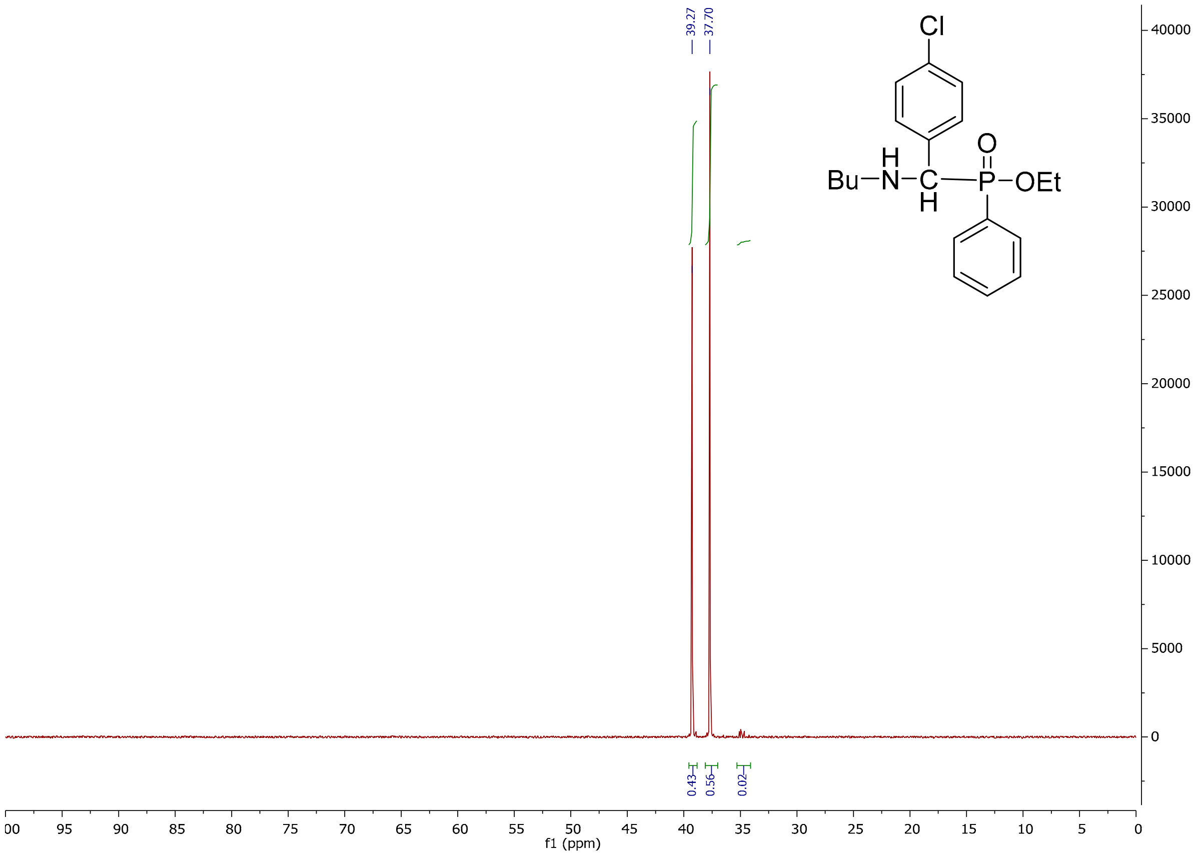

^{13}C {^{1}H} NMR (202 MHz, CDCl$_3$) spectra for 6e

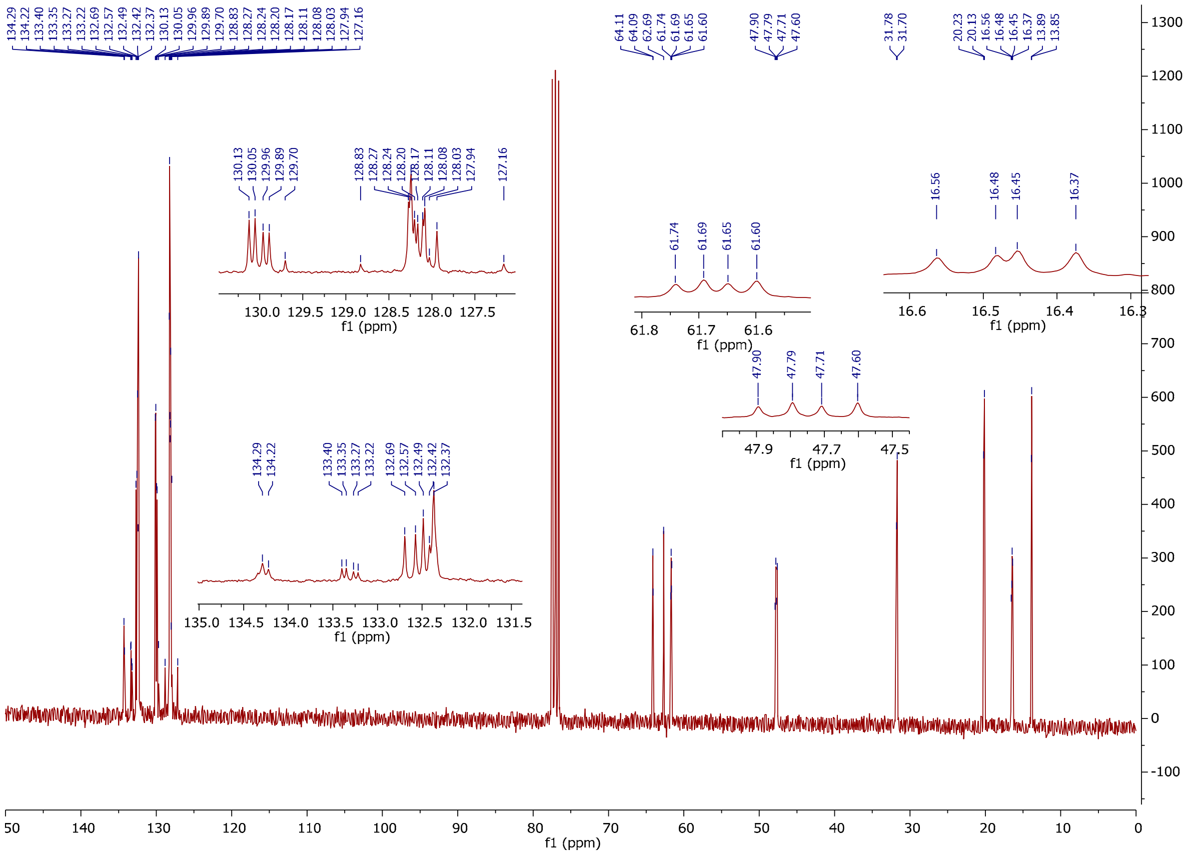

^{1}H NMR (500 MHz, $CDCl_3$) spectra for 6e

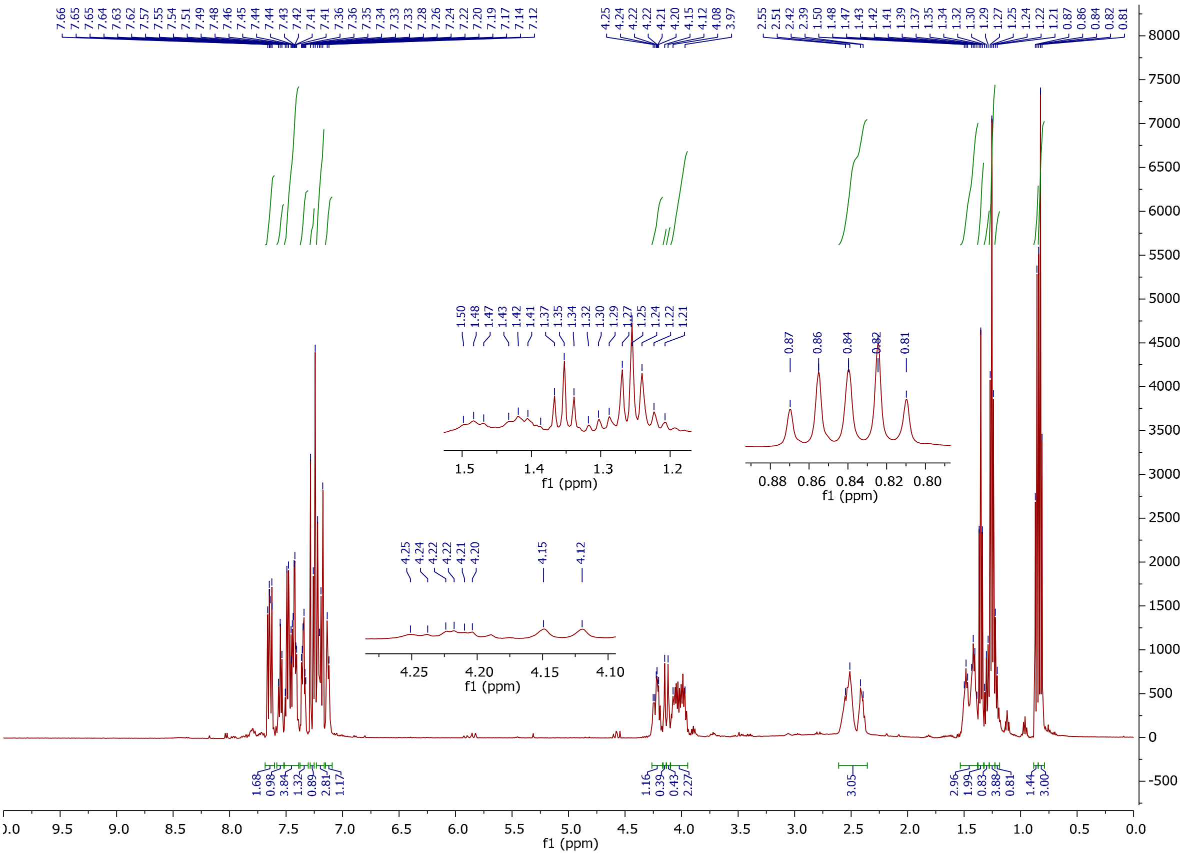

^{31}P {^{1}H} NMR (202 MHz, $CDCl_3$) spectra for 6f

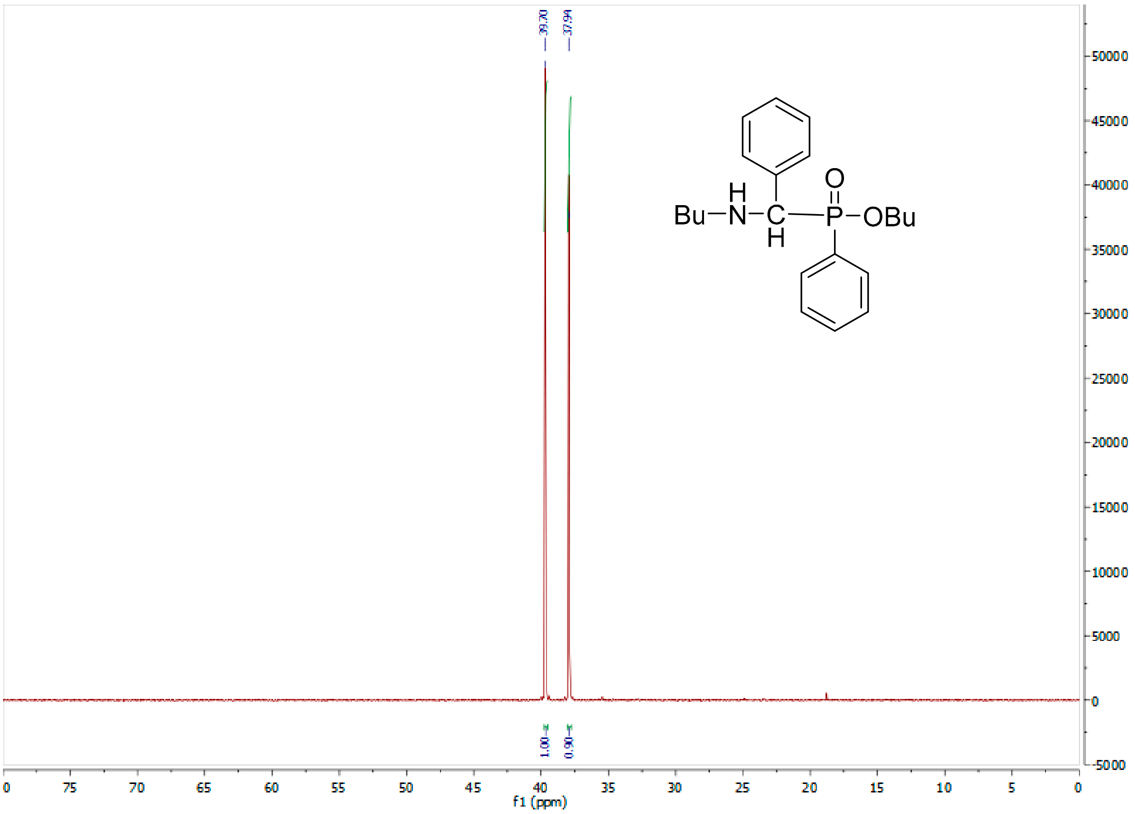

^{13}C {^{1}H} NMR (202 MHz, $CDCl_3$) spectra for 6f

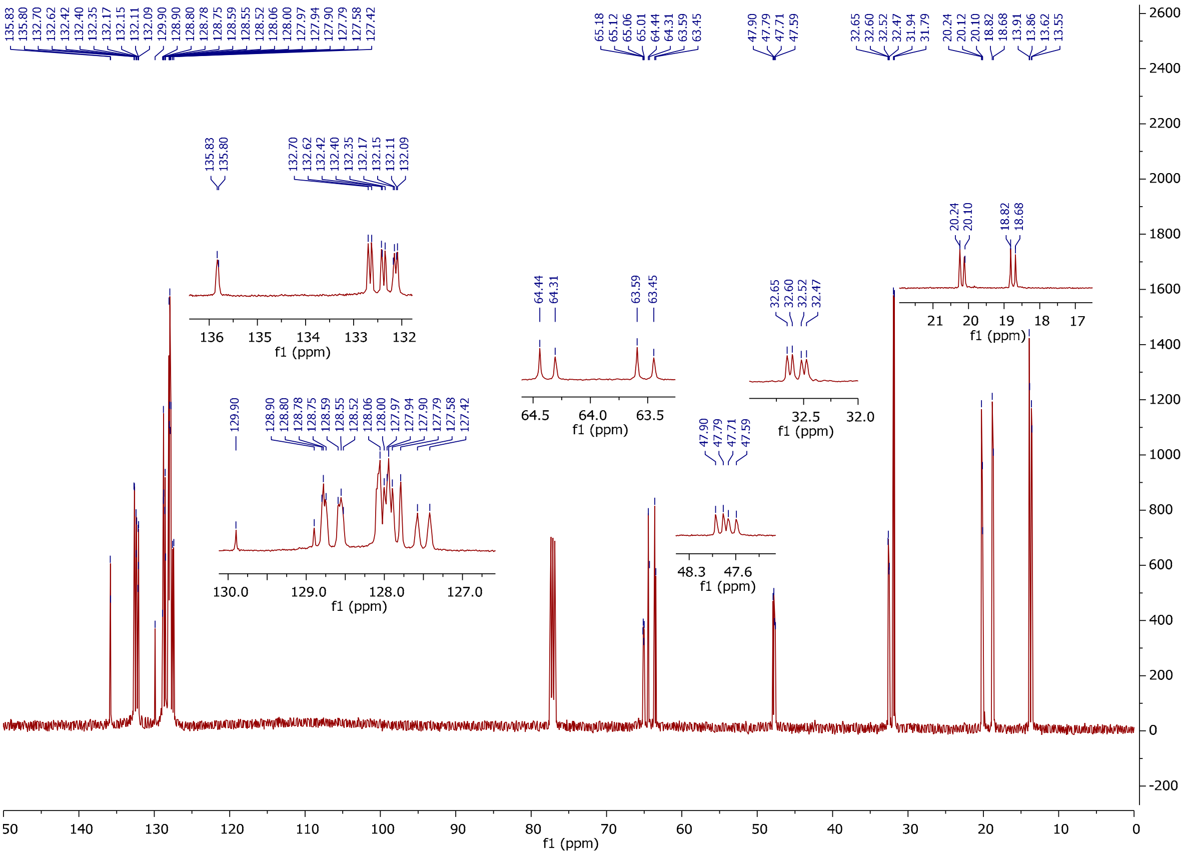

^{1}H NMR (500 MHz, $CDCl_3$) spectra for 6f

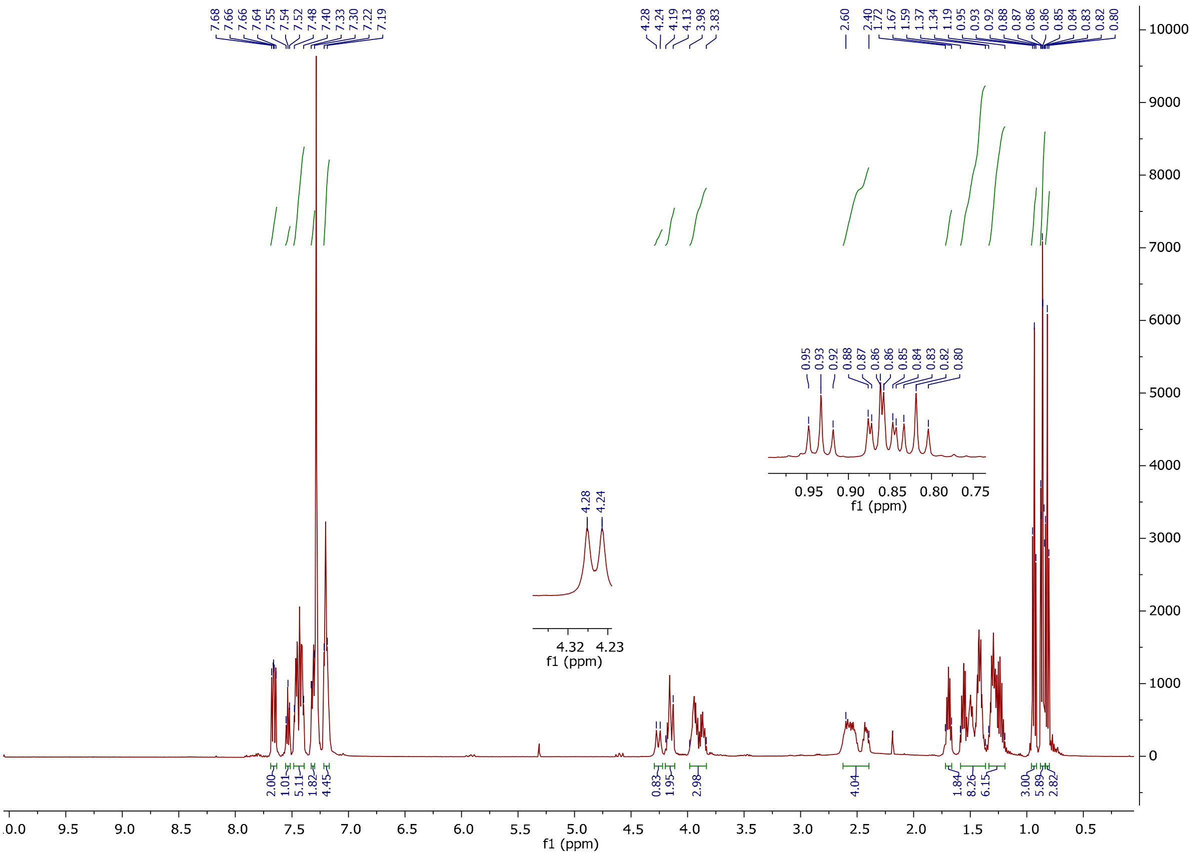

^{31}P {^{1}H} NMR (202 MHz, $CDCl_3$) spectra for 6g

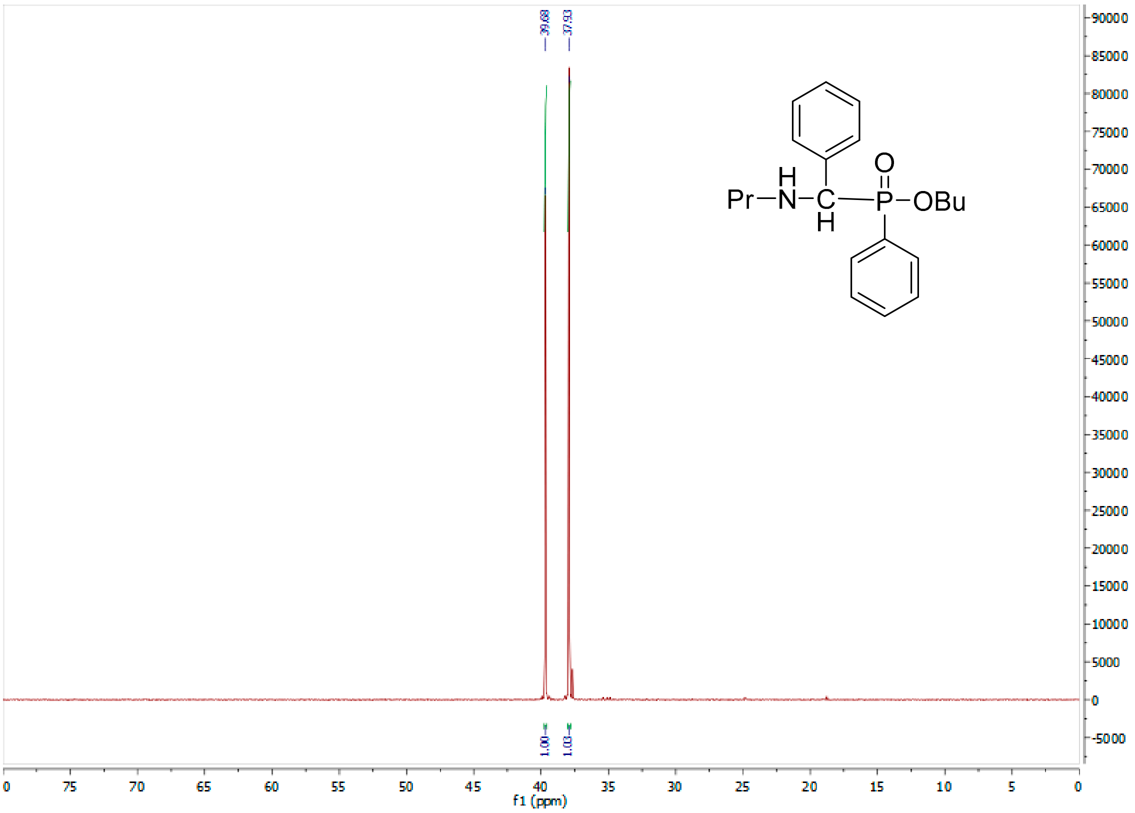

^{13}C {^{1}H} NMR (202 MHz, $CDCl_3$) spectra for 6g

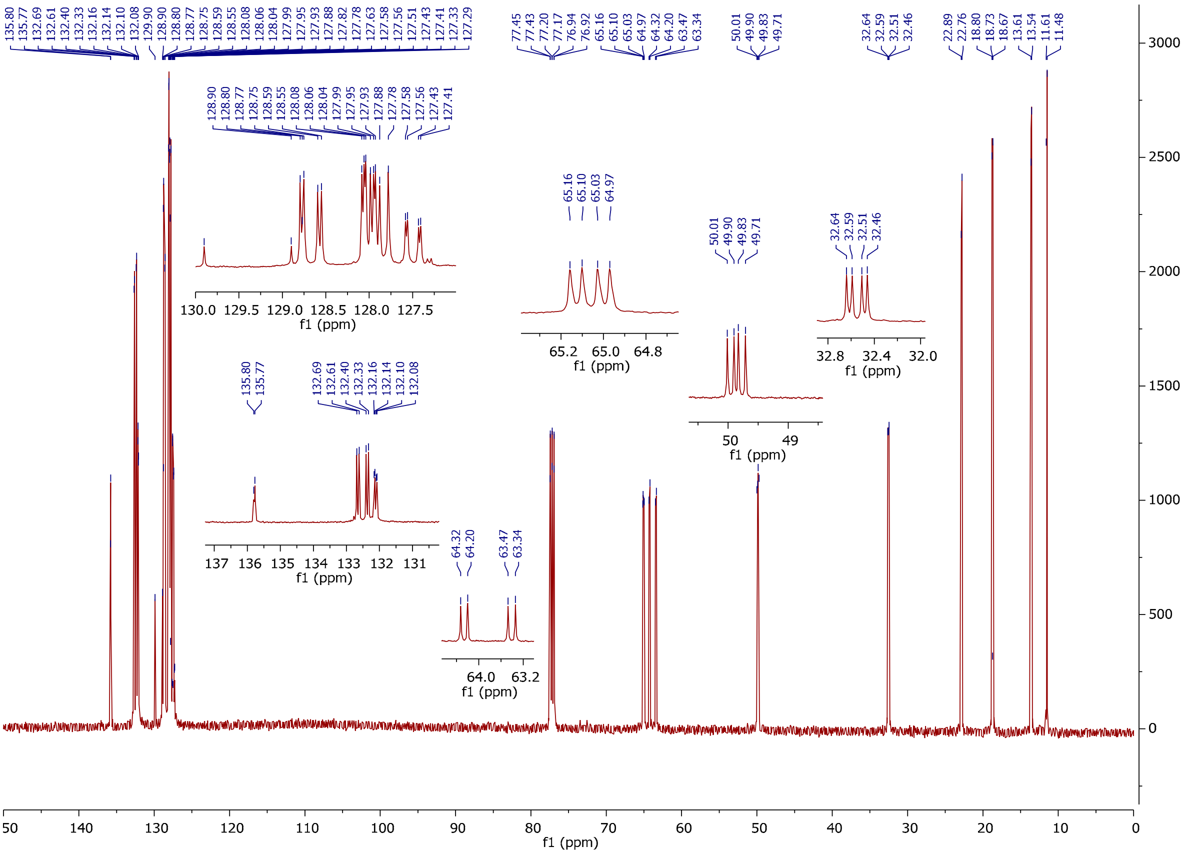

^{1}H NMR (500 MHz, $CDCl_3$) spectra for 6g

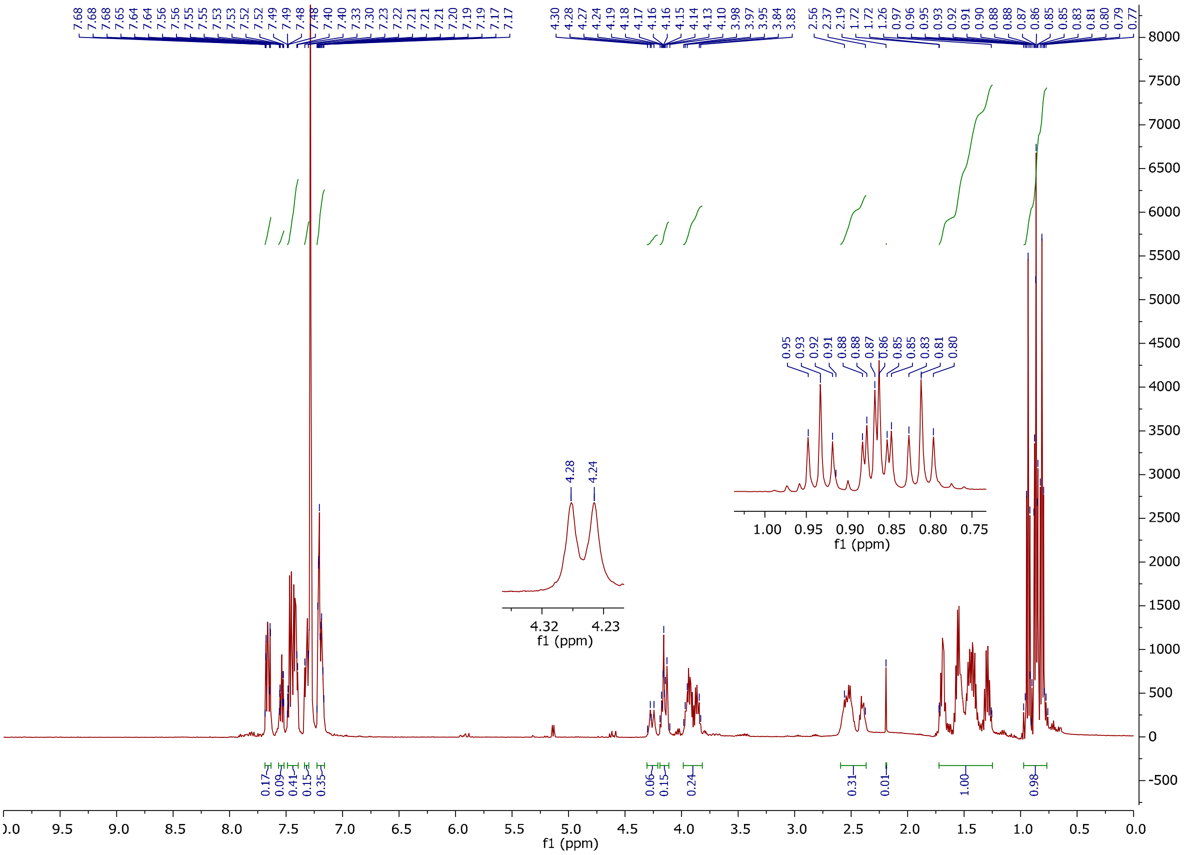

^{31}P {^{1}H} NMR (202 MHz, $CDCl_3$) spectra for 6h

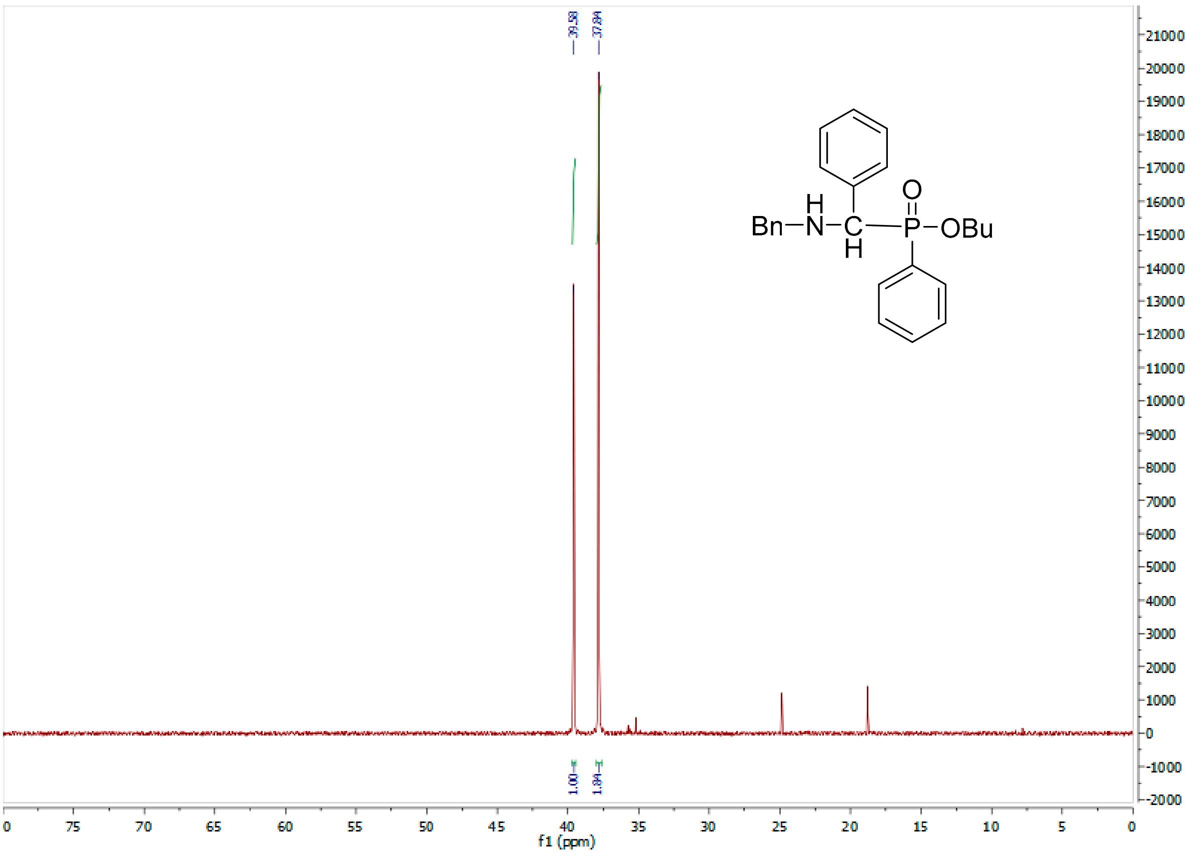

$^{13}C\{^1H\}$ NMR (202 MHz, CDCl$_3$) spectra for 6h

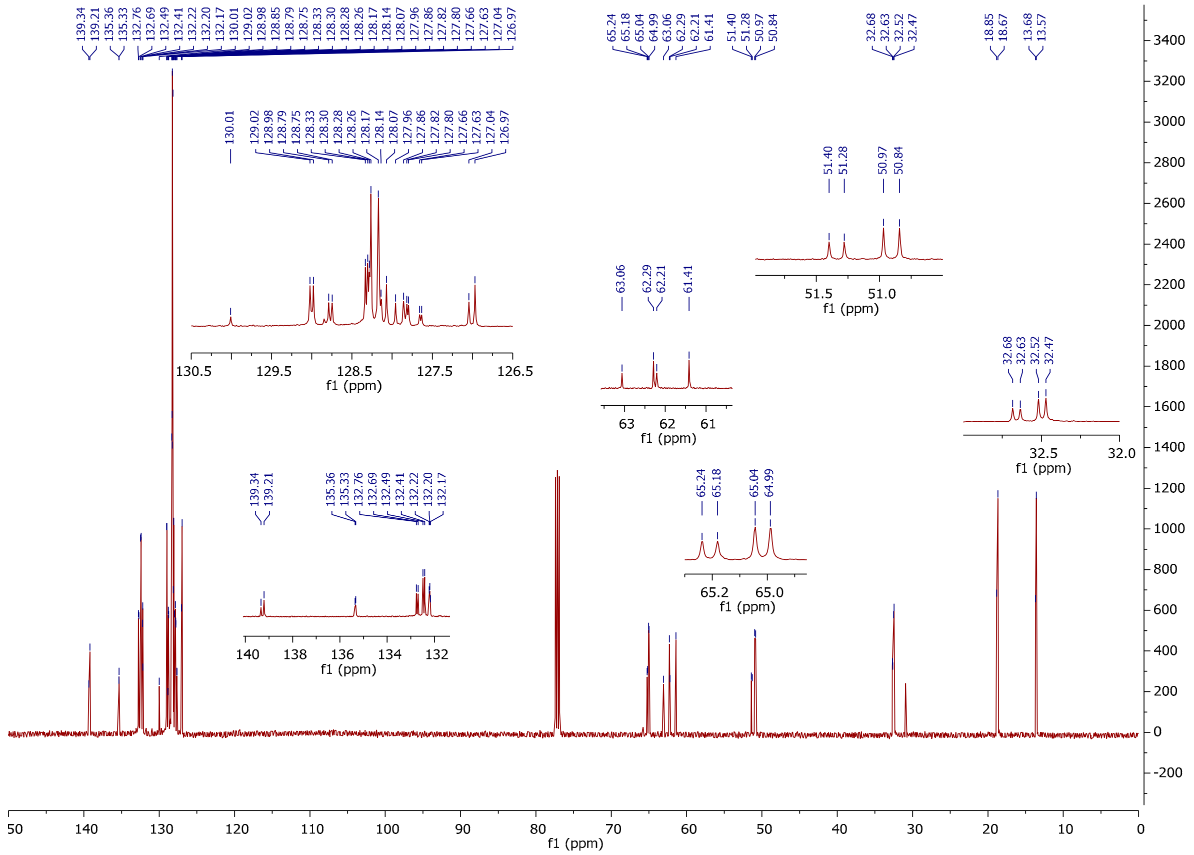

^1H NMR (500 MHz, CDCl$_3$) spectra for 6h

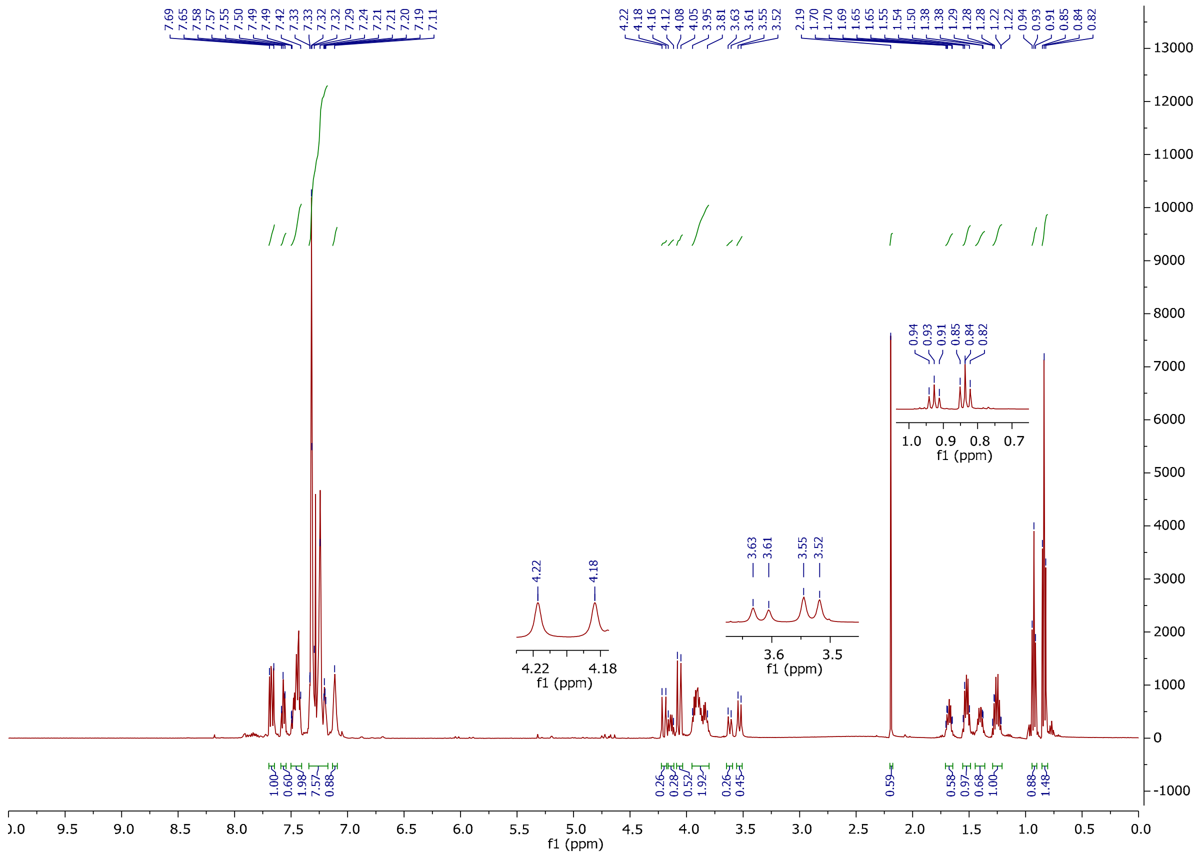

2. HPLC-MS analysis for the compounds 6a-c,e,f,h synthesized

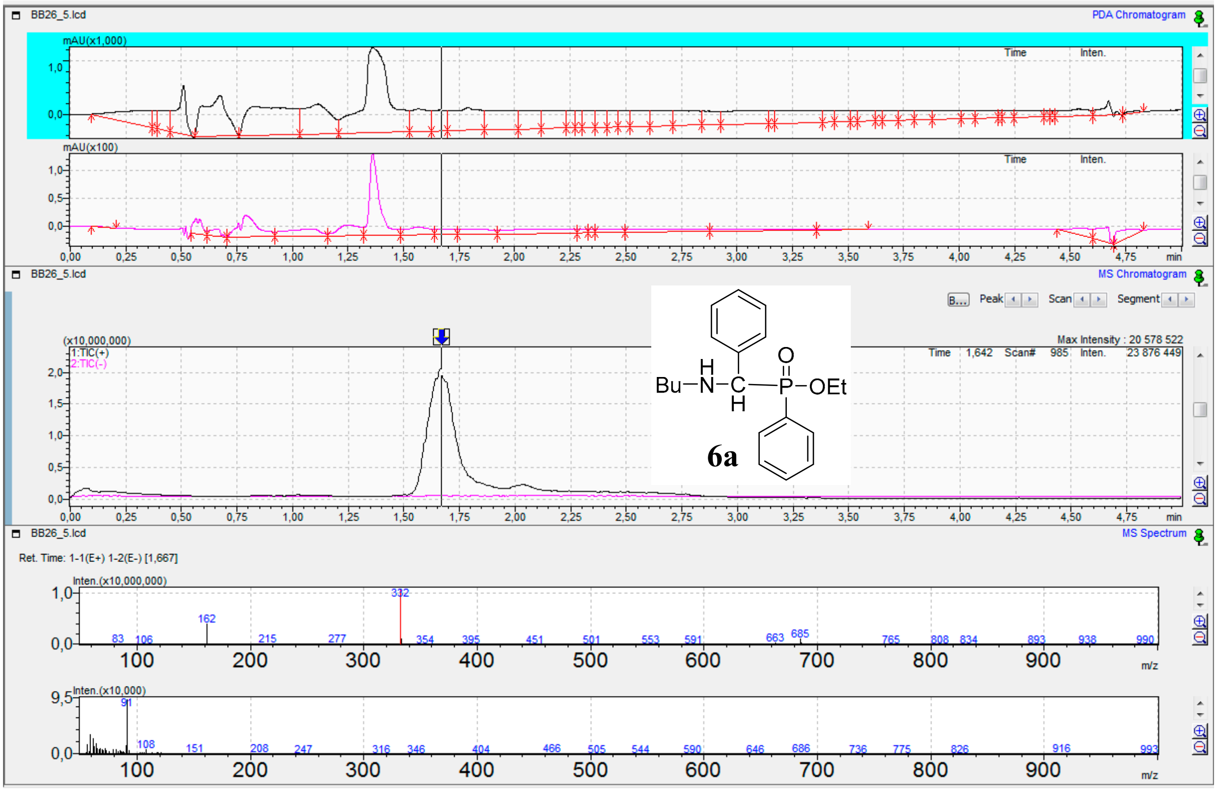

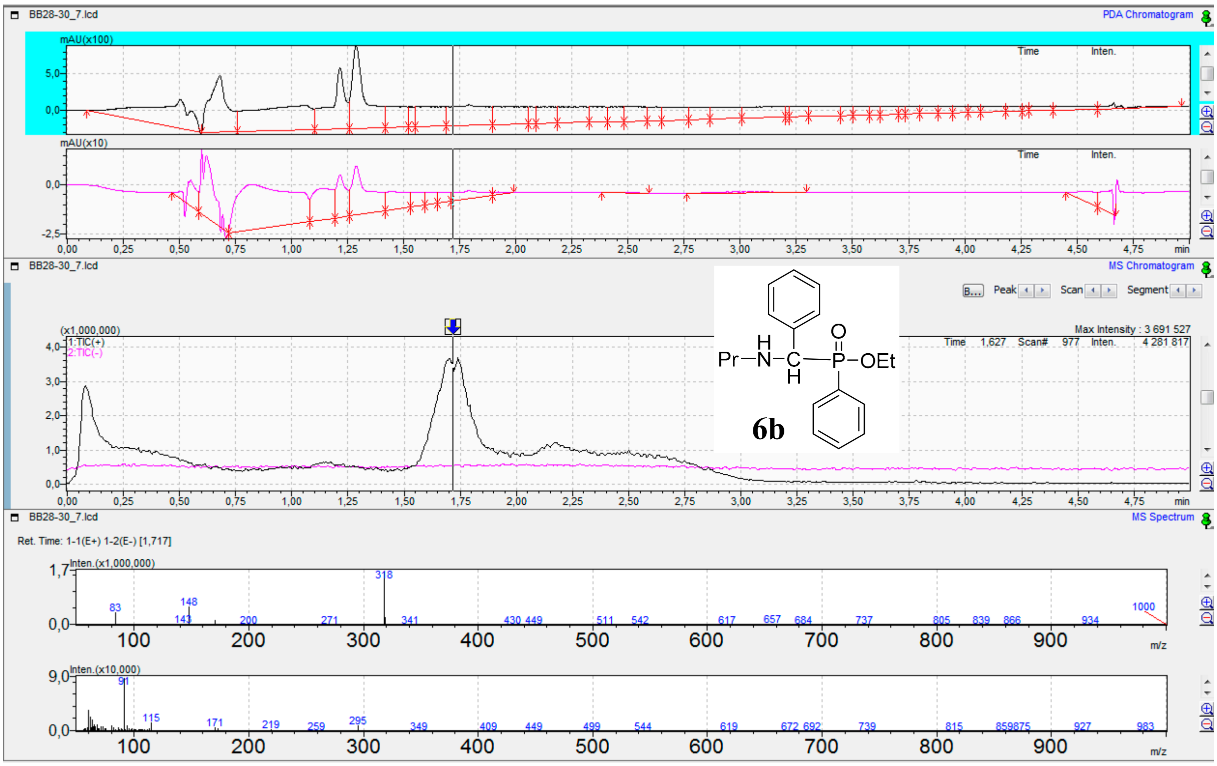

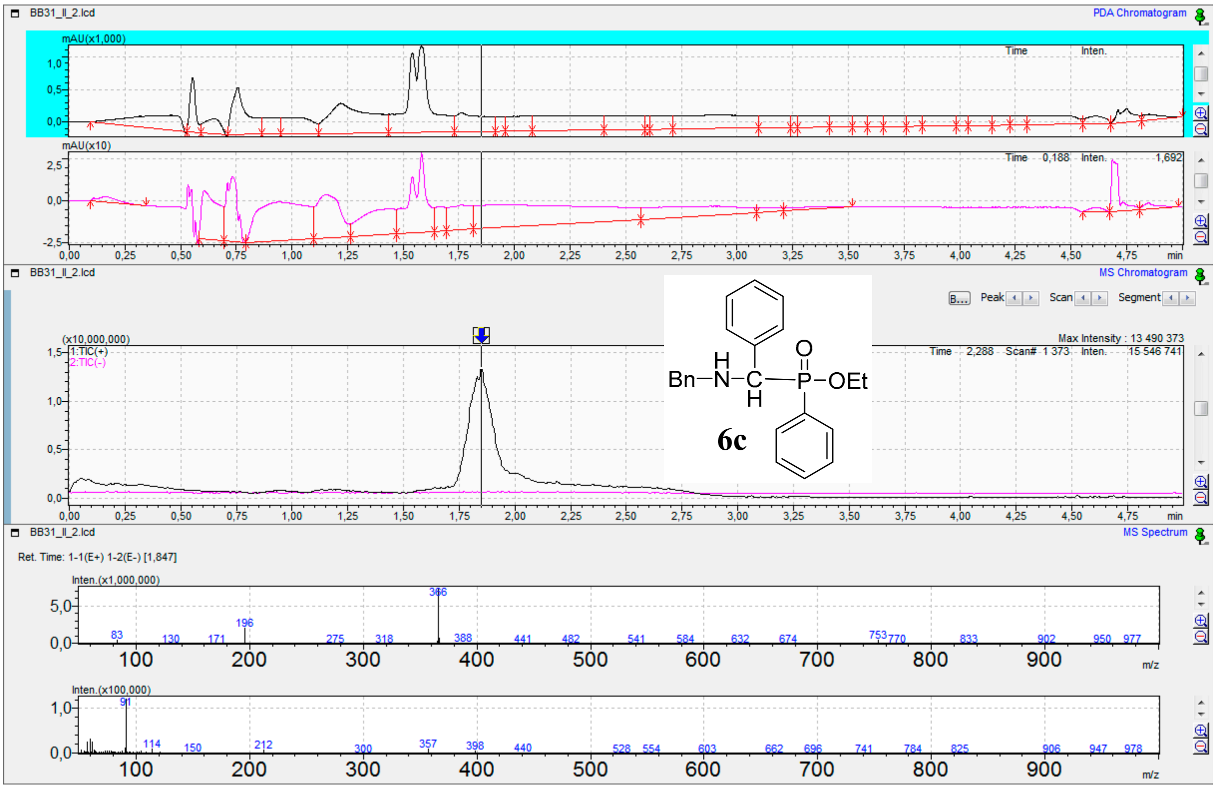

BB31_II_2.lcd
PDA Chromatogram
mAU(x1,000)
mAU(x10)
MS Chromatogram
(x10,000,000)
1:TIC(+)
2:TIC(-)
Max Intensity : 13 490 373
Time 2,288 Scan# 1 373 Inten. 15 546 741
Bn–N(H)–C(H)–P(O)–OEt
6c
MS Spectrum
Ret. Time: 1-1(E+) 1-2(E-) [1,847]
Inten.(x1,000,000)
Inten.(x100,000)
366
196
91
m/z
min

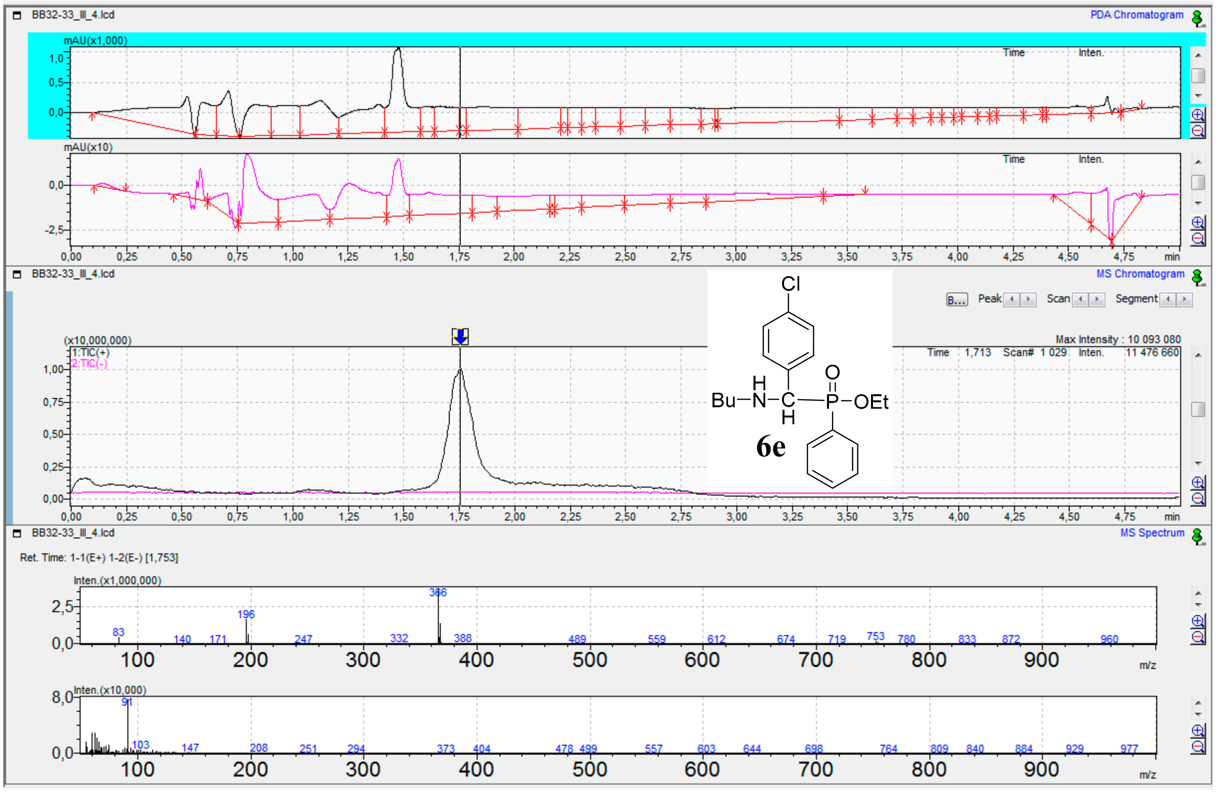

BB32-33_III_4.lcd
PDA Chromatogram
mAU(x1,000)
mAU(x10)
MS Chromatogram
(x10,000,000)
1:TIC(+)
2:TIC(-)
Max Intensity : 10 093 080
Time 1,713 Scan# 1 029 Inten. 11 476 660
Cl
Bu–N(H)–C(H)–P(O)–OEt
6e
MS Spectrum
Ret. Time: 1-1(E+) 1-2(E-) [1,753]
Inten.(x1,000,000)
Inten.(x10,000)
366
196
91
m/z
min

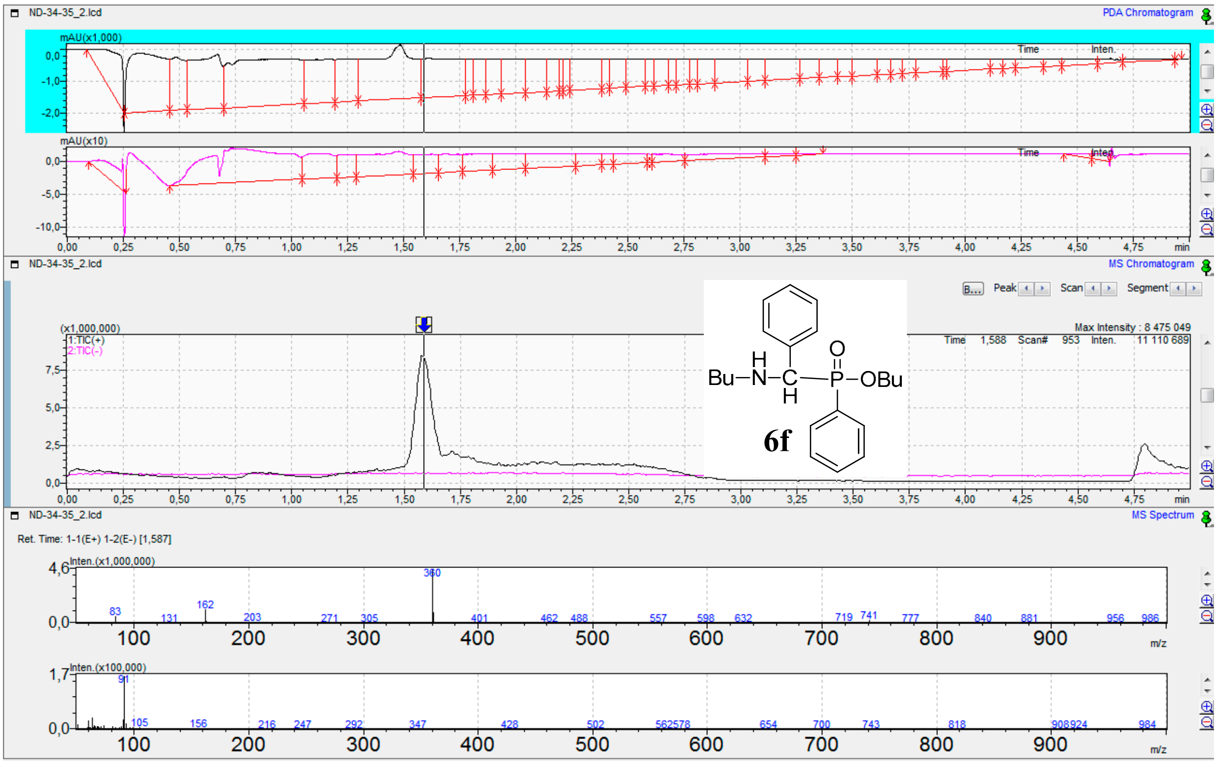
ND-34-35_2.lcd
PDA Chromatogram
MS Chromatogram
MS Spectrum
Max Intensity : 8 475 049
Ret. Time: 1-1(E+) 1-2(E-) [1,587]
1:TIC(+)
2:TIC(-)
Bu–N(H)–C(H)–P(=O)–OBu
6f
m/z
min

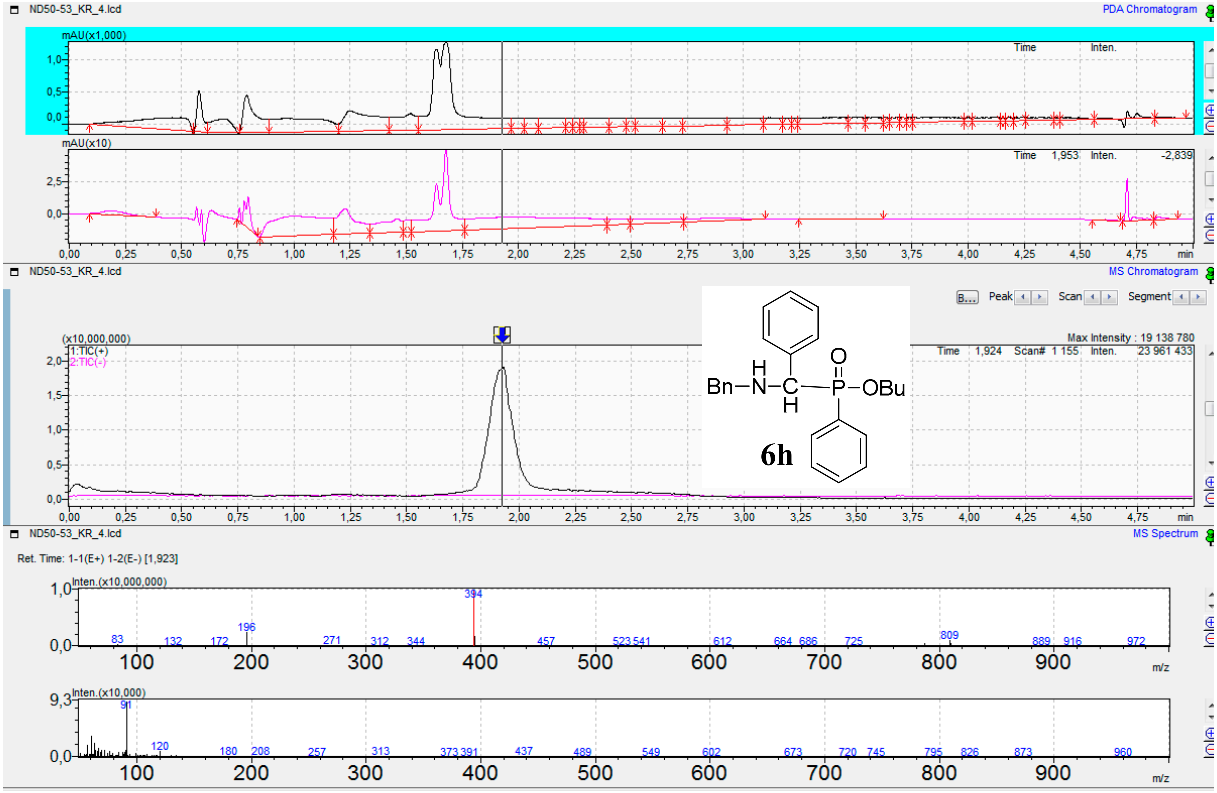
ND50-53_KR_4.lcd
PDA Chromatogram
MS Chromatogram
MS Spectrum
Max Intensity : 19 138 780
Ret. Time: 1-1(E+) 1-2(E-) [1,923]
1:TIC(+)
2:TIC(-)
Bn–N(H)–C(H)–P(=O)–OBu
6h
m/z
min
